# Hierarchical Accumulation of Histone Variant H2A.Z Regulates Transcriptional States and Histone Modifications in Early Mammalian Embryos

**DOI:** 10.1002/advs.202200057

**Published:** 2022-06-19

**Authors:** Xin Liu, Jingjing Zhang, Jilong Zhou, Guowei Bu, Wei Zhu, Hainan He, Qiaoran Sun, Zhisheng Yu, Wenjing Xiong, Liyan Wang, Danya Wu, Chengli Dou, Longtao Yu, Kai Zhou, Shangke Wang, Zhengang Fan, Tingting Wang, Ruifeng Hu, Taotao Hu, Xia Zhang, Yi‐Liang Miao

**Affiliations:** ^1^ Institute of Stem Cell and Regenerative Biology College of Animal Science and Veterinary Medicine Huazhong Agricultural University Wuhan 430070 P. R. China; ^2^ Key Laboratory of Agricultural Animal Genetics Breeding and Reproduction (Huazhong Agricultural University) Ministry of Education Wuhan 430070 P. R. China; ^3^ Hubei Hongshan Laboratory Wuhan 430070 P. R. China

**Keywords:** H2A.Z, histone modification, lineage commitment, preimplantation development, zygotic genome activation

## Abstract

Early embryos undergo extensive epigenetic reprogramming to achieve gamete‐to‐embryo transition, which involves the loading and removal of histone variant H2A.Z on chromatin. However, how does H2A.Z regulate gene expression and histone modifications during preimplantation development remains unrevealed. Here, by using ultra‐low‐input native chromatin immunoprecipitation and sequencing, the genome‐wide distribution of H2A.Z is delineated in mouse oocytes and early embryos. These landscapes indicate that paternal H2A.Z is removed upon fertilization, followed by unbiased accumulation on parental genomes during zygotic genome activation (ZGA). Remarkably, H2A.Z exhibits hierarchical accumulation as different peak types at promoters: promoters with double H2A.Z peaks are colocalized with H3K4me3 and indicate transcriptional activation; promoters with a single H2A.Z peak are more likely to occupy bivalent marks (H3K4me3+H3K27me3) and indicate development gene suppression; promoters with no H2A.Z accumulation exhibit persisting gene silencing in early embryos. Moreover, H2A.Z depletion changes the enrichment of histone modifications and RNA polymerase II binding at promoters, resulting in abnormal gene expression and developmental arrest during lineage commitment. Furthermore, similar transcription and accumulation patterns between mouse and porcine embryos indicate that a dual role of H2A.Z in regulating the epigenome required for proper gene expression is conserved during mammalian preimplantation development.

## Graphical abstract

The authors delineate the H2A.Z landscapes in mouse oocytes and early embryos and unexpectedly identify its two incorporation modes on chromatin during zygotic genome activation as double and single peaks. This hierarchical accumulation of promoter H2A.Z indicates its dual role in inducing the local epigenetic changes for gene expression regulation during preimplantation development.

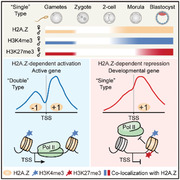

## 1 Introduction

Histones are the main proteins that provide structural support for DNA packaging and epigenetic modifications to control gene expression. Aside from canonical histones (e.g., H2A, H2B, H3.1, H3.2, and H4), which are deposited during DNA replication, non‐canonical histone variants (e.g., H2A.Z, H2A.X, TSH2B, and H3.3) are deposited by histone chaperones and remodeling complexes in a temporally and spatially controlled manner.^[^ ^1^ ^]^ Notably, histone variants can influence DNA accessibility by regulating nucleosome structure and stability, or recruiting variant‐specific interacting proteins to induce local epigenetic changes.^[^ ^1^ ^]^ In the histone H2A family, H2A.Z is involved in multiple nuclear processes including gene regulation,^[^ ^2^ ^]^ chromosomal segregation,^[^ ^3^ ^]^ heterochromatin formation,^[^ ^4^ ^]^ genome integrity,^[^ ^5^ ^]^ and DNA repair.^[^ ^6^ ^]^ By means of chromatin immunoprecipitation and sequencing (ChIP‐seq), H2A.Z has been found to mark nucleosomes across the transcription start sites (TSSs) of most genes in mouse embryonic stem cells (ESCs).^[^ ^2^, ^7^ ^]^ However, conflicting reports show that this promoter H2A.Z exhibits either positive^[^ ^7a,b^ ^]^ or negative^[^ ^2^, ^7^ ^]^ functions for driving gene expression. Therefore, the relationship between H2A.Z and transcriptional states needs further elucidation.

Interestingly, previous studies in ESCs^[^ ^7^, ^8^ ^]^ also indicate that H2A.Z recruits mixed lineage leukemia (MLL) complex (e.g., RBBP5) and polycomb repressive complex 2 (PRC2) (e.g., SUZ12, EZH2) at gene promoters, and thus establishes H3K4me3/H3K27me3 bivalency. Depletion of H2A.Z^[^ ^7^, ^8^ ^]^ or its regulators^[^ ^9^ ^]^ (e.g., YEATS4, INO80) reduces the targeting of PRC2 complex or some pluripotency factors (e.g., POU5F1), results in gene dysregulation and compromises either self‐renewal for ESCs or lineage commitment during differentiation. Remarkably, H2A.Z deficiency also impairs mouse embryonic development around the time of implantation.^[^ ^10^ ^]^ Due to the technical limitation in research methods, so far, H2A.Z dynamics in early embryos is only investigated through immunofluorescence. An intense H2A.Z signal has been found in metaphase II (MII) oocytes, followed by a total removal upon fertilization.^[^ ^11^ ^]^ However, the recovery of H2A.Z signal is reported to be observed at the 2‐cell stage^[^ ^12^ ^]^ or the blastocyst stage^[^ ^11^ ^]^ from different studies.

By using ultra‐low‐input native chromatin immunoprecipitation and sequencing (ULI‐NChIP‐seq),^[^ ^13^ ^]^ direct measurements of epigenetic marks can be achieved with small quantities of cells. The genome‐wide distribution and dynamics of H3K4me3, H3K27me3, and H3K9me3 have been determined in mouse oocytes and early embryos through ULI‐NChIP‐seq.^[^ ^14^ ^]^ In this study, we utilize the same method to delineate the H2A.Z landscape in early mammalian development, and uncover two accumulation modes of promoter H2A.Z during zygotic genome activation (ZGA). We propose that this hierarchical accumulation of H2A.Z shows dual functions to regulate the transcriptional states and histone modifications in early embryos and that precise de novo incorporation of H2A.Z into chromatin is necessary for normal development.

## 2 Results

### 2.1 Genome‐Wide Profiling of H2A.Z in Mouse MII Oocytes and Early Embryos

**Figure 1:**
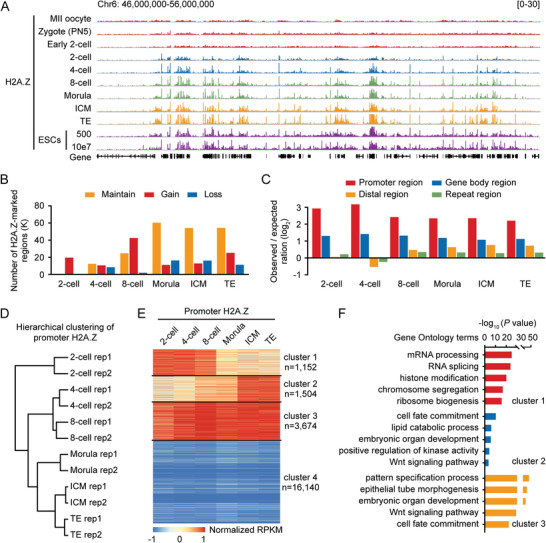
Genome‐wide profiling of H2A.Z in mouse MII oocytes and early embryos. A) Genome browser snapshot of H2A.Z enrichment in mouse metaphase II (MII) oocytes, in vivo‐fertilized (IVO) embryos at the zygote, early 2‐cell, 2‐cell, 4‐cell, 8‐cell and morula stages, the inner cell mass (ICM) and the trophectoderm (TE) of blastocysts, and mouse embryonic stem cells (ESCs) (500 cells, ULI‐NChIP‐seq in this study; 10e7 cells, conventional ChIP‐seq from a previous publication^[^ ^9a^ ^]^). Chr, chromosome; PN5, pronuclear stage 5. Two replicates of H2A.Z ULI‐NChIP‐seq are merged for each indicated stage of embryos. B) Bar charts showing the number of H2A.Z peaks gained, lost, and maintained at each stage by comparing to the previous stage. C) Bar charts showing the enrichment of H2A.Z in promoter, gene body, distal, and repeat regions. Promoter is defined as the ± 1 kb genomic region around transcription start site (TSS). D) Hierarchical clustering of promoter H2A.Z enrichment in early embryos. E) Heatmaps showing the dynamics of H2A.Z enrichment at all gene promoters (*n* = 22470) in early embryos. Genes are clustered into four groups by *k*‐means algorithms, and the numbers of genes in each cluster are shown. RPKM, reads per kilobase of bin per million mapped reads. F) Bar charts showing the enriched Gene Ontology (GO) terms for gene cluster 1–3 in (E).

Through analyzing the public RNA sequencing (RNA‐seq) data^[^ ^15^ ^]^ and quantitative PCR (qPCR), we found that mouse *H2A.Z* mRNA was highly transcribed at the 2‐cell stage, namely the timing for major ZGA, which is correlated with readily detectable H2A.Z protein after the 2‐cell stage by immunofluorescence (Figure S1A–C, Supporting Information). To explore H2A.Z dynamics in early mouse embryos, four commercial antibodies of H2A.Z were tested by ULI‐NChIP‐seq using 31–32 morulae (about 500 cells). The most suitable antibody for ULI‐NChIP‐seq was then determined by analyzing the quality of ChIP‐seq data (Figure S1D,E, Supporting Information). We also performed H2A.Z ULI‐NChIP‐seq using 500 ESCs, and found that the global H2A.Z enrichment was highly correlated with the conventional ChIP‐seq data^[^ ^16^ ^]^ (Figure S1F, Supporting Information). After this method validation, we generated H2A.Z profiles of mouse MII oocytes and in vivo‐fertilized (IVO) embryos from zygotes to blastocysts (separated inner cell mass (ICM) and trophectoderm (TE)) (Table S1, Supporting Information). Notably, H2A.Z exhibited prominent accumulation as sharp peaks after the 2‐cell stage (**Figure** **1**A), and two independent replicates among these samples were highly correlated (Figure S1G, Supporting Information). H2A.Z also exhibited similar distribution in these post‐ZGA embryos when compared with that in mouse ESCs (Figure 1A). To our surprise, MII oocytes showed a strong immunostaining signal but no evident genomic enrichment by ULI‐NChIP‐seq (Figure 1A and Figure S1C, Supporting Information). Given that H2A.Z could be washed away by Triton X‐100 before paraformaldehyde fixation,^[^ ^17^ ^]^ whereas H3K4me3 exhibited a stable immunostaining signal by the same treatment, we speculate that H2A.Z may associate with the metaphase chromosomes but not be incorporated into chromatin (Figure S1H, Supporting Information).

To identify H2A.Z‐enriched regions in early embryos, we performed peak calling using model‐based analysis of ChIP‐seq (MACS),^[^ ^18^ ^]^ and found an increasing number of H2A.Z peaks during preimplantation development (Figure 1B and Figure S2A, Supporting Information). Importantly, H2A.Z preferred to occupy the promoter region, especially for the H2A.Z peaks maintained from the previous stage (Figure 1B,C and Figure S2B,C, Supporting Information). Our hierarchical clustering analysis revealed that promoter H2A.Z accumulation in 2‐ to 8‐cell embryos was distinct from those in morulae and blastocysts (Figure 1D). By using *k*‐means algorithms, promoter H2A.Z accumulation in early embryos was classified into four clusters: cleavage‐specific accumulation (cluster 1), blastocyst‐specific accumulation (cluster 2), all‐stage accumulation (cluster 3), all‐stage no accumulation (cluster 4) (Figure 1E). Gene Ontology (GO) analysis indicated that promoter H2A.Z in cluster 1 was deposited for genes involved in RNA processing, histone modification and chromosome segregation; promoter H2A.Z in cluster 2 and cluster 3 were deposited for genes involved in cell fate commitment, embryonic organ development and Wnt signaling pathway (Figure 1F). These results suggest that promoter H2A.Z accumulation is associated with gene expression for diverse biological functions in early embryos.

### 2.2 H2A.Z Localization in Sperm is not Decisive for H2A.Z Establishment in Early Embryos

**Figure 2:**
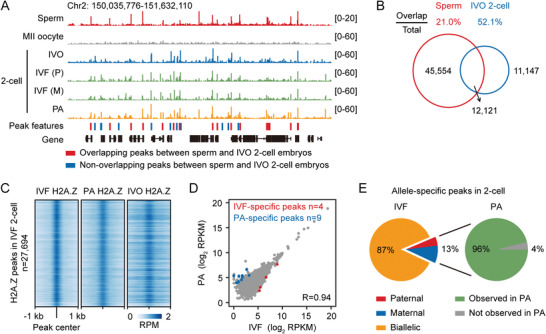
Sperm H2A.Z peaks are not maintained in early embryos. A) Genome browser snapshot of H2A.Z enrichment in mouse sperm (CD1), oocytes (C57BL/6) and 2‐cell embryos from IVO by crossing B6D2F1 females with B6D2F1 males, in vitro fertilization (IVF) by using C57BL/6 oocytes and DBA/2 sperm, and parthenogenetically activation (PA) by using C57BL/6 oocytes. H2A.Z peaks marked in red and blue are overlapping peaks and non‐overlapping peaks between sperm and IVO 2‐cell embryos, respectively. Chr, chromosome; M, maternal; P, paternal. H2A.Z ChIP‐seq data in sperm are from a previous publication.^[^ ^19^ ^]^ Two replicates of H2A.Z ULI‐NChIP‐seq are merged for each sample. B) Venn diagrams showing the overlapping and non‐overlapping H2A.Z peaks between sperm and IVO 2‐cell embryos. Percentages of overlapping peaks in the total peaks of each sample and the numbers of overlapping and non‐overlapping peaks are shown. C) Heatmaps showing the enrichment of all H2A.Z peaks (defined in IVF 2‐cell embryos, n = 27694) in IVF, PA, and IVO 2‐cell embryos. RPM, read counts per million mapped reads. D) Scatter plots comparing the H2A.Z enrichment between IVF and PA 2‐cell embryos. Pearson's correlation coefficient is shown on the bottom‐right panel. The numbers of specific peaks (Fold Change (FC) >2 and false discovery rate <0.05) in each sample are shown on the top panel. E) Pie charts showing the percentages of H2A.Z peaks with paternal, maternal, and biallelic features in IVF 2‐cell embryos (left), and the percentages of these allele‐specific peaks observed or not observed in PA 2‐cell embryos (right).

By performing conventional ChIP‐seq with 10–20 million sperm cells, a recent study reports that mouse sperm genomes are occupied by sharp H2A.Z peaks.^[^ ^19^ ^]^ In this study, we found that almost all H2A.Z peaks (*n* = 57675) in sperm were removed upon fertilization (Figure S2A, Supporting Information). Nevertheless, we noticed that about 21% of H2A.Z peaks in sperm were reestablished at the 2‐cell stage (**Figure** **2**A,B). To determine whether embryonic H2A.Z localization is derived from the paternal genome, we generated H2A.Z profiles of parthenogenetically activated (PA) 2‐cell embryos using C57BL/6 oocytes, which lack a contribution from sperm genome. We also used in vitro fertilization (IVF) to generate 2‐cell embryos in which maternal (C57BL/6) and paternal (DBA/2) chromatin could be distinguished. Here, we found that H2A.Z enrichment in IVF embryos was highly similar to that in PA embryos and IVO embryos (Figure 2A,C), and the numbers of differentially enriched H2A.Z peaks between IVF and PA embryos were <10 for each sample (Figure 2D). We next determined 6202 allele‐specific H2A.Z peaks in IVF 2‐cell embryos. Strikingly, about 87% of these H2A.Z peaks were biallelic in IVF embryos, and almost all (96%) of the remaining allele‐specific H2A.Z peaks were detected in PA embryos (Figure 2E). These data suggest that H2A.Z‐enriched chromatin is established de novo during major ZGA without significant allelic bias on the two parental genomes, and this establishment process is independent of the paternal genome.

### 2.3 Peak Types of Promoter H2A.Z are Associated with Gene Expression and Pol II Binding

**Figure 3:**
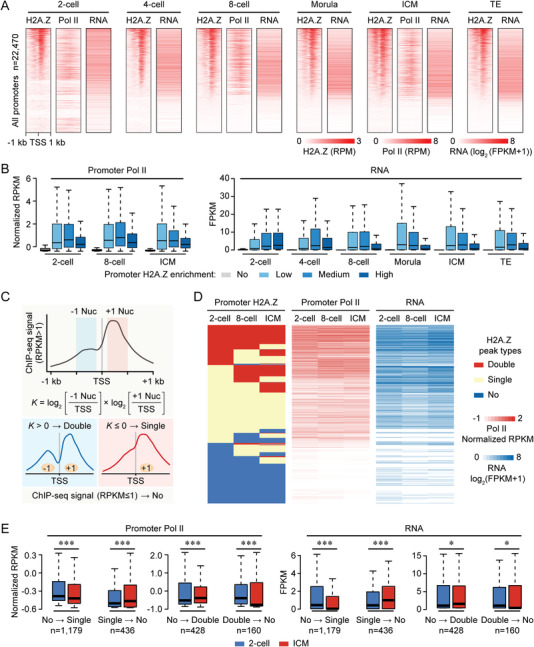
Promoter H2A.Z has dual functions for gene activation and silencing. A) Heatmaps showing the H2A.Z and RNA polymerase II (Pol II) enrichment in early embryos at all gene promoters (*n* = 22470). Each row represents a promoter region (TSS ± 1 kb) and is ordered descending by H2A.Z enrichment. RNA levels at each stage are also shown. Pol II ChIP‐seq and RNA‐seq data for early embryos are from previous publications.^[^ ^15^, ^20^ ^]^ FPKM, fragments per kilobase of transcript per million mapped reads. B) Boxplots showing Pol II enrichment (left) and RNA levels (right) for four gene clusters in early embryos. Genes with weak promoter H2A.Z enrichment (RPKM ≤1) are extracted first, and the remaining genes are divided into three tertiles depending on promoter H2A.Z enrichment. For boxplots, middle lines indicate the median, the boxes indicate the 25^th^/75^th^ percentiles, and the whiskers indicate 1.5× interquartile range (IQR). C) Schematic showing a strategy for the classification of three H2A.Z peak types at promoters. Nuc, nucleosome. D) Heatmaps showing the dynamics of promoter H2A.Z peak types (left), Pol II enrichment (middle) and RNA levels (right) in 2‐cell embryos, 8‐cell embryos and ICM. E) Boxplots showing the changes of promoter Pol II enrichment (left) and RNA levels (right) when H2A.Z peak transition occurs from 2‐cell embryos to ICM. The numbers of genes with different transition modes are shown. ***P <0.001; *P <0.05; two‐sided Wilcoxon‐Mann‐Whitney test. For boxplots, middle lines indicate the median, the boxes indicate the 25^th^/75^th^ percentiles, and the whiskers indicate 1.5× IQR.

We next focused on how promoter H2A.Z impacts gene expression^[^ ^15d^ ^]^ and RNA polymerase II (Pol II) binding^[^ ^20^ ^]^ during preimplantation development. All promoters (*n* = 22470) were then ordered by decreasing H2A.Z signal in early embryos (**Figure** **3**A), or partitioned into four parts based on H2A.Z enrichment (Figure 3B). We found that the highest levels of promoter H2A.Z enrichment did not coincide with the highest levels of gene expression or Pol II signal (Figure 3A,B). Intriguingly, when we classified promoters based on gene expression levels, average profiles of H2A.Z exhibited double peaks (±1 nucleosomes) at active gene promoters (FPKM >1), and exhibited a single peak (+1 nucleosome) at inactive gene promoters (FPKM <0.1) (Figure S3A,B, Supporting Information). More importantly, this positional hierarchy of promoter H2A.Z was not stage‐specific, which was also observed in ESCs.

Accordingly, strong signal of promoter H2A.Z (RPKM >1) was then quantified as *K* values to define the hierarchical accumulation: “Double” type, *K* >0; “Single” type, *K* ≤0. Meanwhile, weak signal of promoter H2A.Z (RPKM ≤1) was termed “No” type (Figure 3C). We then classified all promoters into “Double”, “Single” and “No” type in 2‐cell embryos, 8‐cell embryos and ICM (Table S2, Supporting Information) and found that H2A.Z preferentially occupied CpG‐rich promoters in each sample, where double and single H2A.Z peaks showed no difference in CG content and H2A.Z enrichment (Figure S3C,D, Supporting Information). It suggests that H2A.Z accumulation as “Double” and “Single” types is not associated with promoter CG content. We also eliminated the possibility that our methodology influenced our interpretation of H2A.Z peaks, because both “Double” and “Single” types were detected in our MNase‐based ULI‐NChIP‐seq and sonication‐based ChIP‐seq data^[^ ^9a^ ^]^ (Figure S3E, Supporting Information). Notably, promoters with double H2A.Z peaks showed higher levels of gene expression and Pol II signal when compared to promoters with “Single” and “No” types of H2A.Z (Figure S3D, Supporting Information). Dynamic changes of different H2A.Z peak types in 2‐cell embryos, 8‐cell embryos and ICM also showed similar relative levels for their impacts on gene expression and Pol II binding (Figure 3D). When compared the H2A.Z peak transition between 2‐cell embryos and ICM, we found that the transition from “No” to “Single” type or from “Double” to “No” type was associated with transcriptional silencing and lower Pol II binding, whereas the transition from “No” to “Double” type or from “Single” to “No” type was associated with transcriptional activation and higher Pol II binding (Figure 3E). These data suggest that accumulation types of H2A.Z at gene promoters, but not its intensity, are tightly related to the transcriptional states in early embryos and ESCs.

We then performed motif enrichment analysis to identify putative transcriptional factors (TFs) binding to the H2A.Z‐enriched regions in early embryos. First, we focused on the top 10 TFs enriched at H2A.Z peaks in 2‐cell embryos, 8‐cell embryos and ICM (Figure S4A, Supporting Information). We observed high enrichment of MYC family factors in 2‐cell embryos (two MYC isoforms, MYCN, MAX, and MNT), and high enrichment of EST family factors in ICM (ELF1, ELK4, GABPA, ETS1, and ETV2). Meanwhile, both were enriched in 8‐cell embryos, indicating a regulatory transition of TFs during this stage. The enrichment of MYC motif is in accordance with the observation that MYC activates ribosomal RNA transcription in mouse 2‐cell embryos,^[^ ^21^ ^]^ indicating that H2A.Z incorporation may directly initiate MYC‐mediated ZGA. By contrast, EST family TFs have previously been shown to regulate vascular and hematopoietic embryonic development.^[^ ^22^ ^]^ It suggests that H2A.Z peaks in ICM contribute to initiating the lineage differentiation program, as shown by the GO terms in Figure 1F. We also computed the enrichment of TFs at promoter H2A.Z, and identified some TFs that were more likely to bind “Double” or “Single” H2A.Z peaks (Figure S4B, Supporting Information). For example, the most enriched TFs for “Double” H2A.Z peaks were Sp and NFY family factors, which have also been reported to be ZGA regulators in mouse embryos.^[^ ^23^ ^]^ Additionally, lineage specification factors, like Jun, JunD and MyoD,^[^ ^22^, ^24^ ^]^ were preferentially enriched for “Single” H2A.Z peaks. Overall, our analyses suggest an extensive reprogramming of TFs to drive H2A.Z‐marked gene expression at the preimplantation stages.

### 2.4 Promoter H2A.Z Colocalizes with H3K4me3 and H3K27me3 in Early Embryos

**Figure 4:**
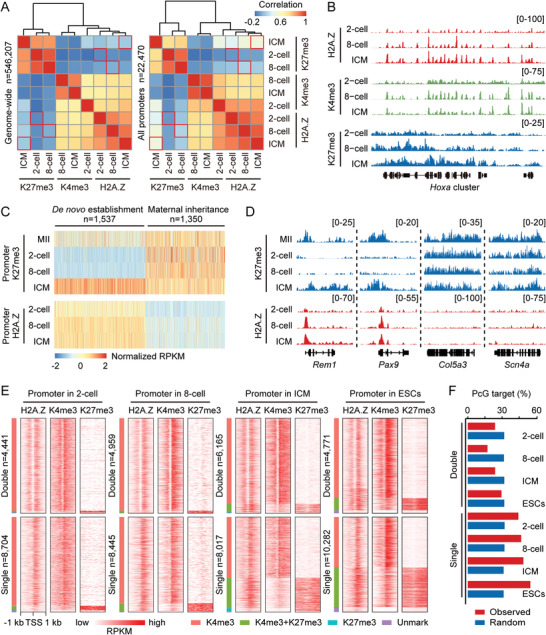
Colocalization among H2A.Z, H3K4me3, and H3K27me3. A) Heatmaps and hierarchical clustering showing the Pearson's correlations among H2A.Z, H3K4me3 (K4me3) and H3K27me3 (K27me3) enrichment in 2‐cell embryos, 8‐cell embryos, and ICM on genome‐wide (5‐kb bins, *n* = 546207, left) and at promoters (*n* = 22470, right). H3K4me3 and H3K27me3 ChIP‐seq data for early embryos are from a previous publication.^[^ ^14a^ ^]^ Red boxes mark the correlations between H2A.Z and H3K27me3 in the same sample. B) Genome browser snapshot of H2A.Z, H3K4me3, and H3K27me3 enrichment in 2‐cell embryos, 8‐cell embryos and ICM near *Hoxa* cluster. C) Heatmaps showing the H2A.Z enrichment in early embryos at promoters with de novo established (*n* = 1537) or maternally inherited (*n* = 1350) H3K27me3 in ICM. D) Genome browser snapshot of H2A.Z and H3K27me3 enrichment in MII oocytes and early embryos near *Rem1*, *Pax9*, *Col5a3*, and *Scn4a*. E) Heatmaps showing the H2A.Z, H3K4me3, and H3K27me3 enrichment in 2‐cell embryos, 8‐cell embryos, ICM and ESCs at promoters with “Double” and “Single” H2A.Z peaks. The numbers of genes with “Double” and “Single” H2A.Z peaks in each sample are shown. Promoters are also divided into four clusters based on their histone modification patterns as follows: H3K4me3 (red), H3K27me3 (cyan), bivalent mark (H3K4me3+H3K27me3, green), and Unmark (purple). F) Percentages of genes with “Double” and “Single” H2A.Z peaks that are polycomb group (PcG) target genes in 2‐cell embryos, 8‐cell embryos, ICM and ESCs. A similar analysis for a set of random genes with the same size to the corresponding genes is performed as control.

Previous studies in ESCs revealed that H2A.Z colocalized with H3K4me3 at active promoters and colocalized with bivalent marks (H3K4me3+H3K27me3) at poised promoters.^[^ ^7^, ^9^ ^]^ We then explored a potential relationship among H2A.Z, H3K4me3, and H3K27me3 accumulation^[^ ^14a^ ^]^ in early embryos, and found that H2A.Z strongly correlated with H3K4me3 and anti‐correlated with H3K27me3 both on genome‐wide and at promoters (**Figure** **4**A,B). First, previous studies have demonstrated that broad and flat H3K4me3 peaks become narrow and sharp at the 2‐cell stage, and this H3K4me3 peak transition is considered to benefit major ZGA initiation in mouse embryos.^[^ ^14^, ^15^ ^]^ Here, H2A.Z started to incorporate into chromatin at the same 2‐cell stage, and colocalized with sharp H3K4me3 peaks in the subsequent stages (Figure 4B). Given that H2A.Z‐enriched genes showed higher expression levels than genes with no H2A.Z enrichment (Figure S3E, Supporting Information), these results raise the possibility that H2A.Z incorporation may contribute to ZGA initiation at the 2‐cell stage. Second, the correlation between H2A.Z and H3K27me3 became more positive in ICM when compared to their correlations in cleavage embryos (Figure 4A and Figure S5A, Supporting Information). Promoter H3K27me3 enrichment in ICM was then divided into two parts: de novo establishment at the blastocyst stage; inheritance from MII oocyte genomes. To our surprise, H2A.Z tended to prematurely occupy promoters with de novo H3K27me3 established in ICM, but not to occupy promoters with maternally inherited H3K27me3 (Figure 4C,D). This finding implies that H2A.Z plays a prerequisite role in H3K27me3 establishment during blastocyst formation, in which the first lineage commitment occurs.

We then tested whether different H2A.Z peak types were occupied by different histone modifications (Figure 4E and Table S2, Supporting Information). First, the majority of promoters with “Double” and “Single” H2A.Z peaks only colocalized with H3K4me3 at the 2‐cell (H3K4me3‐marked gene: “Double” type, *n* = 4318; “Single” type, *n* = 8087) and 8‐cell stages (H3K4me3‐marked gene: “Double” type, *n* = 4816; “Single” type, *n* = 7649). Second, along with lineage commitment, some H2A.Z‐enriched promoters acquired H3K27me3 modification to form H3K4me3/H3K27me3 bivalency (Bivalent gene: 2‐cell embryos, *n* = 442; 8‐cell embryos, *n* = 824; ICM, *n* = 3126; ESCs, *n* = 4579). Strikingly, these bivalent marks preferentially occupied promoters with “Single” rather than “Double” H2A.Z peaks in early embryos and ESCs (Bivalent gene: “Double” type, *n* = 71, 122, 550, and 464 in each sample; “Single” type, *n* = 371, 702, 2576, and 4114 in each sample) (Figure 4E). Coincidently, the proportion of bivalent genes, defined by polycomb‐group (PcG) protein targets in ESCs,^[^ ^25^ ^]^ was higher in genes with “Single” than “Double” H2A.Z peaks during preimplantation development (Figure 4F). We thus hypothesize that H2A.Z serves as a platform to facilitate the binding of MLL complex and PRC2 complex for H3K4me3 and H3K27me3 deposition in early embryos, as previously reported in ESCs.^[^ ^7^, ^9^ ^]^

Intriguingly, we noticed that H3K4me3 also accumulated as “Double” and “Single” peaks in line with the corresponding H2A.Z‐enriched promoters (Figure 4E). To examine if H3K4me3 possesses similar regulatory features as H2A.Z, we performed same analyses of promoter H3K4me3 in 2‐cell embryos, 8‐cell embryos and ICM. After partitioning all promoters into four parts based on H3K4me3 enrichment, we found that the highest levels of promoter H3K4me3 enrichment coincided with the highest levels of gene expression or Pol II signal (Figure S5B, Supporting Information). We also classified promoter H3K4me3 peaks as “Double”, “Single” and “No” types (Figure S5C, Supporting Information) using the same strategy for H2A.Z (Figure 3C). Similar to H2A.Z peaks, the H3K4me3 intensities of “Double” and “Single” peaks were comparable in mouse embryos (Figure S5C, Supporting Information). However, unlike H2A.Z that exhibited different gene regulations between their “Double” and “Single” types (Figure 3E), “Double” and “Single” peaks of H3K4me3 exhibited no difference for gene expression or Pol II signal (Figure S5C, Supporting Information). These findings suggest that the supportive functions of H3K4me3 for gene expression is strongly associated with its signal intensity, which is different from H2A.Z.

### 2.5 H2A.Z Deficiency Impedes Lineage Commitment in Early Embryos, but Not ZGA

**Figure 5:**
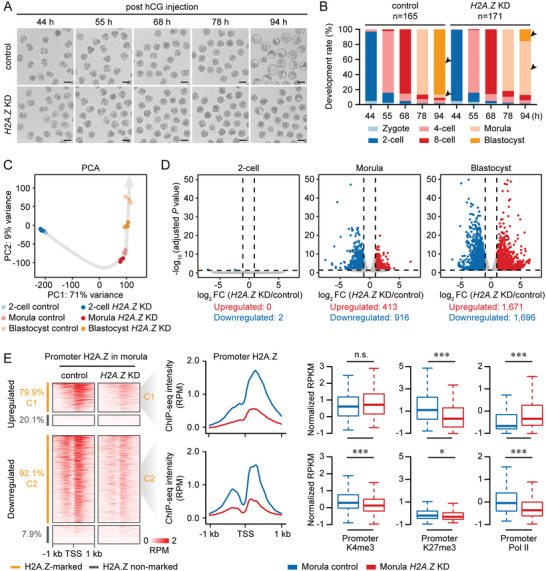
H2A.Z deficiency causes early embryonic arrest with defective transcription and histone modifications. A) Representative images of control and *H2A.Z* knockdown (KD) embryos at the indicated time points post hCG injection. One representative image from five independent experiments is shown. Scale bar, 100 µm. B) Bar charts showing the development rate of control and *H2A.Z* KD embryos at the indicated time points post hCG injection. The numbers of embryos analyzed from three independent experiments are shown. Arrows point out the developmental differences between control and *H2A.Z* KD embryos. C) Principal component analysis of RNA‐seq data from control and *H2A.Z* KD embryos at the 2‐cell, morula and blastocyst stages. 3–4 replicates of RNA‐seq are generated using control and *H2A.Z* KD embryos. D) Volcano plots comparing differentially expressed genes (DEGs) between control and *H2A.Z* KD embryos at the 2‐cell, morula and blastocyst stages. The criteria for upregulated DEGs are log_2_ FC >1, adjusted *P* value <0.05; downregulated DEGs are log_2_ FC <−1, adjusted *P* value <0.05. The numbers of upregulated and downregulated DEGs are shown. E) Heatmaps (left) showing the promoter H2A.Z enrichment of DEGs between control and *H2A.Z* KD morulae. Each row represents a promoter region and is ordered descending by H2A.Z enrichment. DEGs are classified into H2A.Z‐marked upregulated genes (C1, *n* = 330), H2A.Z‐marked downregulated genes (C2, *n* = 844), and nonmarked genes. Profiles (middle) showing the promoter H2A.Z enrichment of C1 and C2 in control and *H2A.Z* KD morulae. Boxplots (right) showing the promoter H3K4me3, H3K27me3, and Pol II enrichment of C1 and C2 in control and *H2A.Z* KD morulae. Two replicates of H2A.Z, H3K4me3, H3K27me3, and Pol II ULI‐NChIP‐seq are generated using control and *H2A.Z* KD morulae. ***P <0.001; *P <0.05; n.s., no significance; two‐sided Wilcoxon‐Mann‐Whitney test. For boxplots, middle lines indicate the median, the boxes indicate the 25^th^/75^th^ percentiles, and the whiskers indicate 1.5× IQR.

To examine whether loss of H2A.Z affects transcriptional states and histone modifications in early embryos, we first needed to determine the developmental phenotype after H2A.Z deficiency. *H2A.Z* mRNA was then targeted by small interfering RNA (siRNA) injection into zygotes (Table S5, Supporting Information), and this approach efficiently depleted mRNA and protein levels of H2A.Z in preimplantation embryos at the 2‐cell, morula and blastocyst stages (Figure S6A–C, Supporting Information). Subsequently, we found that *H2A.Z* knockdown (KD) significantly reduced blastocyst formation (*H2A.Z* KD, 15.8%; control, 86.7%), but had no effect on the development of the morula stage (**Figure** **5**A,B). Furthermore, *H2A.Z* KD impaired the derivation of ESCs and trophoblast stem cells (TSCs) from blastocysts, but had no effect on the total cell numbers in morulae or blastocysts (Figure S6D–F, Supporting Information).

By performing RNA‐seq of control and *H2A.Z* KD embryos, we identified 1329 differentially expressed genes (DEGs) in morulae and 3367 DEGs in blastocysts (Figure 5C,D and Table S3, Supporting Information). GO analysis indicated that upregulated DEGs at the morula and blastocyst stages were involved in cell cycle arrest, apoptotic signaling pathway and embryonic organ development, whereas downregulated DEGs at the morula and blastocyst stages were enriched for RNA process and metabolic process (Figure S6G, Supporting Information). Moreover, *H2A.Z* KD embryos did not exhibit developmental defects at the 2‐cell stage, the expression levels of ZGA genes^[^ ^26^ ^]^ were not influenced by *H2A.Z* KD, and only two DEGs were observed in *H2A.Z* KD 2‐cell embryos (Figure 5D and Figure S7A–C, Supporting Information), we thus concluded that H2A.Z accumulation during major ZGA was not essential for ZGA initiation.

Next, we surprisedly found that the majority (79.9%) of genes upregulated by *H2A.Z* KD (C1) were enriched with “Single” H2A.Z peaks at their promoters, whereas most (92.1%) downregulated genes (C2) were associated with “Double” H2A.Z peaks (Figure 5E and Table S3, Supporting Information). These H2A.Z‐marked genes (C1 and C2) could be regarded as H2A.Z responsive genes when H2A.Z was depleted in morulae. Consistent with the transcriptional states between two types of H2A.Z‐enriched promoters, upregulated genes in C1 had lower expression levels (Figure S5D, Supporting Information) and Pol II signal (Figure 5F) than downregulated genes in C2 in normal control morulae. In addition, H2A.Z responsive genes upregulated by *H2A.Z* KD were more likely to be the PcG target genes (Figure S5E, Supporting Information) enriched for bivalent marks, in line with their GO annotations as development genes. We next performed ULI‐NChIP‐seq of H2A.Z, H3K4me3, H3K27me3, and Pol II using *H2A.Z* KD morulae and found that promoter H2A.Z enrichment of genes in C1 and C2 was dramatically decreased, regardless of H2A.Z peak types (Figure 5E and Figure S5F, Supporting Information). More importantly, H3K4me3 and Pol II signal was only reduced at downregulated responsive gene promoters, whereas the H3K27me3 signal was largely reduced at upregulated responsive gene promoters with enhanced Pol II enrichment (Figure 5E and Figure S5F, Supporting Information). These findings indicate that H2A.Z harbors dual functions to facilitate H3K4me3 and H3K27me3 deposition at both active and poised promoters in early embryos, and H2A.Z depletion disrupts this balance and leads to developmental arrest at the morula stage, namely the beginning of lineage commitment.

To add further support that H2A.Z is required for proper lineage commitment, we performed embryo transfer of control and *H2A.Z* KD zygotes. A previous study has reported that homozygous knockout of *H2A.Z* results in early embryonic lethality at day 5.5‐6.5 post‐coitus.^[^ ^10^ ^]^ We thus obtained the total RNA of 5.5‐day embryos to examine development gene expression. It was hard to collect 5.5‐day *H2A.Z* KD embryos, because its implantation rate (8.9%) was nearly a quarter of that in control group (36.4%) (Figure S6H, Supporting Information). Additionally, H2A.Z deficiency increased the expression of representative development genes, such as *Gata6*, *Foxa2* (endoderm), *T* (mesoderm), *Pax6*, *Neurog1* (ectoderm), and *Hox* cluster gene *Hoxb13* (Figure S6I, Supporting Information). These transcriptional changes are partially coincident with the qPCR results of embryoid body differentiated from H2A.Z‐depleted ESCs,^[^ ^7^, ^9^ ^]^ confirming that H2A.Z maintains H3K27me3 at poised promoters to repress development genes during early embryogenesis.

### 2.6 SRCAP and ANP32E are Essential for Precise H2A.Z Accumulation in Early Embryos

**Figure 6:**
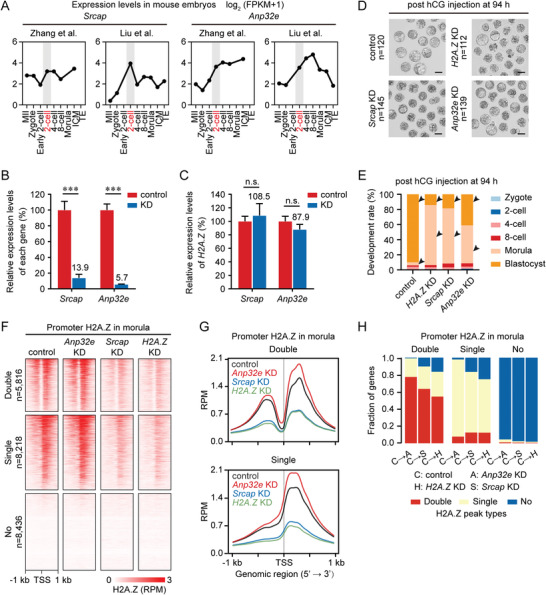
SRCAP and ANP32E deficiency impact precise H2A.Z accumulation in early embryos. A) Line graphs showing the expression levels of *Srcap* and *Anp32e* in mouse MII oocytes and early embryos by RNA‐seq. RNA‐seq data are from previous publications.^[^ ^15c^ ^,d]^ Developmental stage marked in red and gray shades indicates the corresponding zygotic genome activation (ZGA) stage. B) Bar charts showing the relative expression levels of *Srcap* and *Anp32e* in control and KD embryos at the morula stage. Gene expression levels in control embryos are set as 100%, and the relative expression levels in KD embryos are shown as percentages. Error bars represent the standard deviation (SD) in three replicates. ***P <0.001; two‐sided Student's *t*‐test. C) Bar charts showing the relative expression levels of *H2A.Z* in control, *Srcap* KD, and *Anp32e* KD embryos at the morula stage. *H2A.Z* expression levels in control embryos are set as 100%, and the relative *H2A.Z* expression levels in *Srcap* KD and *Anp32e* KD embryos are shown as percentages. Error bars represent the SD in three replicates. n.s., no significance; two‐sided Student's *t*‐test. D) Representative images of control, *H2A.Z* KD, *Srcap* KD, and *Anp32e* KD embryos at 94 h post hCG injection. One representative image and the numbers of embryos analyzed from three independent experiments are shown. Scale bar, 100 µm. E) Bar charts showing the development rate of control, *H2A.Z* KD, *Srcap* KD, and *Anp32e* KD embryos at 94 h post hCG injection. Arrows point out the developmental differences between control and KD embryos. F) Heatmaps showing the H2A.Z enrichment at promoters with “Double”, “Single” and “No” H2A.Z peaks in control, *H2A.Z* KD, *Srcap* KD, and *Anp32e* KD embryos at the morula stage. The numbers of genes with “Double”, “Single” and “No” H2A.Z peaks in control morulae are shown. Each row represents a promoter region and is ordered descending by H2A.Z enrichment. Two replicates of H2A.Z ULI‐NChIP‐seq are generated for each sample. G) Profiles showing the average promoter H2A.Z enrichment with “Double” (top) and “Single” (bottom) peaks in control, *H2A.Z* KD, *Srcap* KD, and *Anp32e* KD embryos at the morula stage. H) Bar charts showing the fractions of genes with different peak transition modes in *H2A.Z* KD, *Srcap* KD, and *Anp32e* KD morulae when compared to control morulae.

In mammalian cells, chromatin remodeler SRCAP^[^ ^27^ ^]^ and molecular chaperone ANP32E^[^ ^28^ ^]^ are known regulators for H2A.Z deposition and eviction from nucleosomes, respectively. Here, we found that both *Srcap* and *Anp32e* were highly transcribed at the 2‐cell stage in the public RNA‐seq data (**Figure** **6**A),^[^ ^15c,d^ ^]^ consistent with the timing for *H2A.Z* transcription (Figure S1A,B, Supporting Information). It suggests that H2A.Z deposition and eviction are two synergetic biological processes when major ZGA occurs. Additionally, KD of *Srcap* and *Anp32e*, with over 85% interference efficiency for each gene (Figure 6B and Table S5, Supporting Information), had no direct effect on *H2A.Z* expression (Figure 6C). Similar to the *H2A.Z* KD experiment, both *Srcap* KD and *Anp32e* KD impaired blastocyst formation, but had no effect on development to the morula stage (Figure 6D,E). In detail, *Srcap* KD (18.4%) exhibited a closer decreased blastocyst rate to *H2A.Z* KD (13.7%) when compared to *Anp32e* KD (40.7%). These observations are consistent with the knockout phenotypes in recent studies, which declare that SRCAP deficiency causes embryonic lethality at the blastocyst stage,^[^ ^29^ ^]^ whereas ANP32E deficiency shows no apparent abnormality.^[^ ^30^ ^]^

By performing ULI‐NChIP‐seq using *Srcap* KD and *Anp32e* KD morulae, we next investigated how SRCAP and ANP32E contribute to H2A.Z accumulation in early embryos. In line with their opposite functions for H2A.Z accumulation, *Anp32e* KD resulted in increased H2A.Z intensity at promoters, whereas *Srcap* KD resulted in decreased H2A.Z intensity at promoters like *H2A.Z* KD (Figure 6F,G). Moreover, when compared to control morulae, KD of *H2A.Z*, *Srcap* and *Anp32e* also led to the peak type transition of H2A.Z at some gene promoters (Figure 6H and Table S4, Supporting Information). *H2A.Z* KD and *Srcap* KD morulae showed similar percentages of promoter H2A.Z transition from “Double” to “Single” (29.0%, 26.2%), “Double” to “No” (16.5%, 10.3%), “Single” to “Double” (12.4%, 12.5%), and “Single” to “No” (25.2%, 16.7%), whereas these percentages were distinct in *Anp32e* KD morulae (21.7%, 0.8%, 7.8%, 2.2%). These data suggest that precise H2A.Z accumulation regulated by SRCAP and ANP32E, especially for SRCAP‐mediated H2A.Z deposition, is vital for preimplantation development.

### 2.7 A Conserved Function of H2A.Z in Mammalian Early Embryos

**Figure 7:**
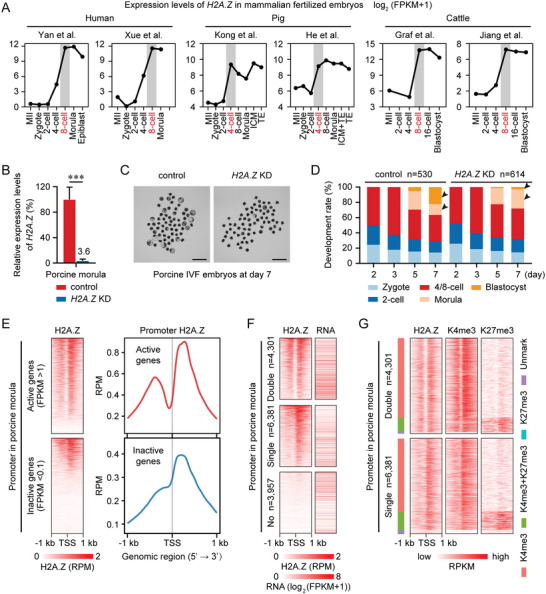
H2A.Z exhibits a conserved function in porcine early embryos. A) Line graphs showing the expression levels of *H2A.Z* in human, porcine and bovine MII oocytes and early embryos by RNA‐seq. RNA‐seq data for human,^[^ ^15^, ^32^ ^]^ pig^[^ ^33^ ^]^ and cattle^[^ ^34^ ^]^ are from previous publications. Developmental stage marked in red and gray shades indicates the corresponding ZGA stage. B) Bar charts showing the relative expression levels of *H2A.Z* in control and *H2A.Z* KD porcine embryos at the morula stage. These embryos are derived by IVF. *H2A.Z* expression levels in control morulae are set as 100%, and the relative *H2A.Z* expression levels in *H2A.Z* KD morulae are shown as a percentage. Error bars represent the SD in 4–5 replicates. ***P <0.001; two‐sided Student's *t*‐test. C) Representative images of control and *H2A.Z* KD porcine embryos at day 7 after fertilization. One representative image from four independent experiments is shown. Scale bar, 500 µm. D) Bar charts showing the development rate of control and *H2A.Z* KD porcine embryos at days 2, 3, 5, and 7 after fertilization. The numbers of embryos analyzed from four independent experiments are shown. Arrows point out the developmental differences between control and *H2A.Z* KD embryos. E) Heatmaps (left) and profiles (right) showing the H2A.Z enrichment at active (top) and inactive (bottom) gene promoters in porcine IVF embryos at the morula stage. Each row represents a promoter region (TSS ± 1 kb) and is ordered descending by H2A.Z enrichment. Two replicates of H2A.Z ULI‐NChIP‐seq are generated using porcine morulae. RNA‐seq data for porcine morulae are from an unpublished report (Genome Sequence Archive accession number: CRA004237). F) Heatmaps showing the H2A.Z enrichment and RNA levels at promoters with “Double”, “Single” and “No” H2A.Z peaks in porcine morulae. The numbers of genes with “Double”, “Single” and “No” H2A.Z peaks in porcine morulae are shown. Each row represents a promoter region and is ordered descending by H2A.Z enrichment. G) Heatmaps showing the enrichment of H2A.Z, H3K4me3, and H3K27me3 at promoters with “Double” and “Single” H2A.Z peaks in porcine morulae. Promoters are also divided into four clusters based on their histone modification patterns as follows: H3K4me3 (red), H3K27me3 (cyan), bivalent mark (H3K4me3+H3K27me3, green) and Unmark (purple). H3K4me3 and H3K27me3 ChIP‐seq data for porcine morulae are from an unpublished report (Genome Sequence Archive accession number: CRA003606). Two replicates of H3K4me3 and H3K27me3 ULI‐NChIP‐seq are generated using porcine morulae.

H2A.Z is one of the most conserved histone variants. It has a monophyletic origin when compared to all histone H2A proteins and variants known to date,^[^ ^31^ ^]^ and shares higher similarity of nucleotide sequence and amino acid sequence in different mammals (Figure S8A,B, Supporting Information). By summarizing the public RNA‐seq data, we also noticed that *H2A.Z* underwent a similar timing for transcriptional activation during ZGA in human,^[^ ^15^, ^32^ ^]^ porcine,^[^ ^33^ ^]^ bovine,^[^ ^34^ ^]^ rat,^[^ ^35^ ^]^ monkey,^[^ ^36^ ^]^ and caprine^[^ ^37^ ^]^ fertilized embryos (**Figure** **7**A and Figure S8C, Supporting Information). These observations strongly suggest that H2A.Z plays a conserved role in mammalian early embryos.

To examine its function during porcine preimplantation development, we injected porcine zygotes with siRNA targeting *H2AFZ*, with over 95% interference efficiency (Figure 7B and Table S5, Supporting Information), and obtained a reduction in blastocyst formation (*H2A.Z* KD, 2.9%; control, 22.5%) (Figure 7C,D), similar to that observed in mice. By performing RNA‐seq and ULI‐NChIP‐seq of H2A.Z, H3K4me3, and H3K27me3 for porcine morulae, we also determined that active gene promoters (FPKM >1) were occupied by “Double” H2A.Z signal, whereas inactive gene promoters (FPKM <0.1) were occupied by “Single” H2A.Z signal (Figure 7E). In accordance with our findings in mice, gene promoters with double H2A.Z peaks exhibited higher expression levels than those with a single H2A.Z peak (Figure 7F). Moreover, almost all promoters with “Double” and “Single” H2A.Z peaks colocalized with H3K4me3 in porcine morulae, and the promoters with “Single” H2A.Z peaks were more likely to deposit H3K27me3 to form bivalency (Figure 7G). These results indicate that the hierarchical accumulation of H2A.Z is a conserved process in early mammalian embryos.

## 3 Discussion

**Figure 8:**
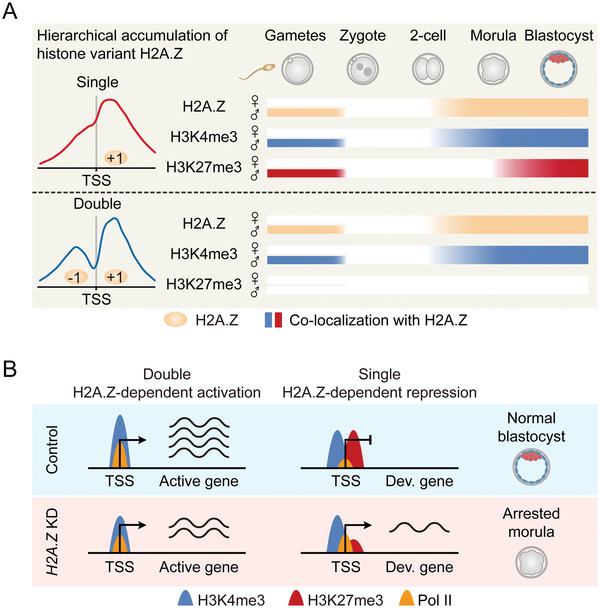
Models of the hierarchical accumulation and function of H2A.Z in early embryos. A) A schematic model showing the reprogramming of H2A.Z from gametes to early embryos. H2A.Z accumulates as “Single” and “Double” peak types at gene promoters in sperm, but exhibits no enrichment in MII oocytes. After fertilization, H2A.Z is globally removed from paternal genome, followed by an unbiased and hierarchical accumulation on parental genomes when *H2A.Z* is highly transcribed during major ZGA (2‐cell stage). Meanwhile, H3K4me3 accumulates with both “Single” and “Double” H2A.Z peaks in sperm and embryos after ZGA, whereas H3K27me3 prefers to accumulate with “Single” H2A.Z peaks in sperm and embryos at the beginning of lineage commitment (morula stage). B) A schematic model showing the different functions for hierarchical H2A.Z accumulation in early embryos. In normal embryos, “Double” H2A.Z peaks only co‐localize with H3K4me3 at promoters, which facilitate Pol II binding and gene activation. Meanwhile, “Single” H2A.Z peaks colocalize with bivalent marks at promoters, which inhibit Pol II binding and poise expression of development genes (Dev. gene). In *H2A.Z* KD embryos, decreased H2A.Z accumulation with “Double” peaks is associated with decreased H3K4me3 and Pol II enrichment at promoters, which downregulates active gene expression. Meanwhile, decreased H2A.Z accumulation with “Single” peaks is associated with decreased H3K27me3 enrichment at promoters, which upregulates development gene expression. This dysregulated transcriptional state finally leads to embryonic arrest at the morula stage.

How histone variants are reprogrammed on genome‐wide scale during mammalian preimplantation development remains largely uncovered. Here, we revealed the dynamics of H2A.Z in mouse oocytes and embryos. H2A.Z shows no accumulation on the maternal genome, whereas paternal H2A.Z is removed upon fertilization, followed by an unbiased accumulation on parental genomes during ZGA (**Figure** **8**A). One major finding of our study is the hierarchical accumulation of H2A.Z as “Double” or “Single” peak types in early embryos, which has not been reported before. These two H2A.Z accumulation modes represent its dual functions to regulate both active and poised genes through establishing different histone modifications at their promoters (Figure 8B). Furthermore, H2A.Z deficiency results in gene dysregulation and embryonic arrest during early lineage commitment (Figure 8B).

As previous described in mouse ESCs,^[^ ^7^, ^9^ ^]^ our data also showed that promoter H2A.Z played dual roles in gene activation and gene silencing during preimplantation development. However, the mechanism underlying its impact on transcription remains a subject of debate in cells. Some studies concluded that H2A.Z recruited Pol II to promoters and reduced the high‐energy barrier to Pol II at +1 nucleosome, which promoted Pol II progression and transcriptional elongation,^[^ ^38^ ^]^ but other reports demonstrated that H2A.Z potentiated Pol II stalling at +1 nucleosome and repressed gene expression.^[^ ^2^ ^]^ In the current study, we conceptualized a novel explanation for two kinds of transcriptional states regulated by H2A.Z: “Double” H2A.Z promoters were found to be active with higher enrichment of Pol II, whereas “Single” H2A.Z promoters were poised with less Pol II signal. Given that these two types of promoters exhibited comparable H2A.Z enrichment, we speculate that the additional H2A.Z peaks at −1 nucleosome possess higher ability for Pol II recruitment, and gene silencing at “Single” H2A.Z promoters is due to Pol II stalling at +1 nucleosome. Nevertheless, we cannot address if the +1 nucleosome in “Double” H2A.Z promoters also shows anchoring of Pol II. Besides, considering that genomic Pol II occupation occurs earlier (1‐cell stage)^[^ ^20^ ^]^ than H2A.Z accumulation (2‐cell stage) in mouse embryos, we propose that H2A.Z is not required for Pol II recruitment on zygotic genomes, but will regulate Pol II kinetics during and after ZGA.

Another explanation for the dual roles of promoter H2A.Z is the local epigenetic marks. In mouse ESCs, H2A.Z accumulates at active promoters, which is colocalized with H3K4me3, and accumulates at poised promoters enriched with bivalent marks (H3K4me3+H3K27me3).^[^ ^7^, ^9^ ^]^ In our study, we further demonstrated that bivalent marks preferentially occupy “Single” H2A.Z promoters in ICM and ESCs, suggesting that “Double” and “Single” H2A.Z promoters exhibit distinct affinities of PRC2 complex for H3K27me3 establishment. The posttranscriptional modifications of H2A.Z may explain this difference, because H2A.Z monoubiquitination has been revealed to stimulate PRC2 recruitment.^[^ ^7^, ^39^ ^]^ It will be interesting in future studies to determine the composition of ubiquitylated H2A.Z between “Double” and “Single” H2A.Z promoters. Moreover, in *H2A.Z* KD embryos, we observed a concomitant reduction of H3K4me3 and H2A.Z at “Double” promoters from downregulated genes, and a concomitant reduction of H3K27me3 and H2A.Z at “Single” promoters from upregulated genes. These results further confirm that the positional hierarchy of H2A.Z provides different promoter environments to influence local epigenome and gene expression in early embryos.

In this study, we found that the accumulation types of H2A.Z were decided at the beginning of nucleosome formation during ZGA. However, how H2A.Z accumulates as “Double” or “Single” peak types is still elusive. Besides the direct KD experiment on H2A.Z, we also depleted its regulators, SRCAP and ANP32E, to examine if these factors influenced H2A.Z accumulation modes. Unexpectedly, we found that KD of *Srcap* and *Anp32e* mainly changed the enrichment of H2A.Z, rather than changing the accumulation types. At present, two studies have reported controversial descriptions in *Anp32e* knockout mouse embryonic fibroblasts (MEFs).^[^ ^7^, ^28^ ^]^ One is consistent with our findings that *Anp32e* deficiency only increases H2A.Z enrichment,^[^ ^28^ ^]^ but another study demonstrates that *Anp32e* knockout results in H2A.Z accumulation at −1 nucleosome when +1 nucleosome has already occupied by H2A.Z,^[^ ^7a^ ^]^ namely promoting the transition from “Single” type to “Double” type. However, this study does not address how ANP32E preferentially evicts H2A.Z at −1 nucleosome. In yeast, the chromatin remodeler SWR1 (an ortholog for SRCAP) preferentially binds long nucleosome‐free DNA (>50 bp) at TSSs and engages both ±1 nucleosomes to deposit double H2A.Z peaks, whereas low‐affinity binding of SWR1 only engages the +1 nucleosome to deposit a single H2A.Z peak.^[^ ^40^ ^]^ These findings suggest that the length of nucleosome‐free DNA and its local accessibility impacts the accumulation types of promoter H2A.Z. Given that both active and inactive promoters are bound by Pol II at the 1‐cell stage,^[^ ^20^ ^]^ we presume that promoters are opened by some prerequisite factors before ZGA initiation. These unknown factors may determine the length of nucleosome‐free DNA at promoters, and thus determine future H2A.Z accumulation at the 2‐cell stage.

Another major finding of this study is the conserved activation of H2A.Z expression during ZGA in different mammalian embryos. Comparably, by ULI‐NChIP‐seq, we found that H2A.Z did not preexist on genomes until ZGA initiation during mouse preimplantation development. Some ZGA‐initiated factors (e.g., SP1, DUX, and NYFA)^[^ ^23^, ^41^ ^]^ have been identified to bind H2A.Z‐enriched promoters in MEFs.^[^ ^7a^ ^]^ In this study, we also observed high enrichment of SP and NFY motifs at H2A.Z‐enriched promoters in 2‐cell embryos. More importantly, when compared to non‐marked promoters, H2A.Z‐marked promoters had high levels of Pol II enrichment and gene expression during mouse ZGA. These findings suggest that H2A.Z provides a potential platform for recruiting TFs to initiate ZGA. However, our subsequent results showed that *H2A.Z* KD experiment had no influence on ZGA gene expression in mice, and had no influence on the development for mouse and porcine embryos to achieve and beyond the ZGA stage. This is consistent with the developmental phenotype of H2A.Z knockout embryos in mice, which exhibits embryonic lethality at postimplantation stage, but not the 2‐cell stage.^[^ ^10^ ^]^

Notably, recent studies have declared that H2A.Z is an important ZGA regulator in *Drosophila melanogaster* ^[^ ^42^ ^]^ and zebrafish embryos.^[^ ^43^ ^]^ In *Drosophila* embryos, ZGA genes contain development genes. The majority of these genes are enriched for H2A.Z at their promoters, and a loss of H2A.Z enrichment through Domino (an ortholog for SRCAP) KD experiment reduces housekeeping gene expression during *Drosophila* ZGA.^[^ ^42^ ^]^ In zebrafish embryos, H2A.Z is incorporated into nucleosomes by SRCAP complex, followed by H3K4me3 establishment at housekeeping genes and bivalency establishment at development genes during ZGA. In addition, *anp32e* (an ortholog for ANP32E) knockout causes premature activation of development genes during zebrafish ZGA.^[^ ^43^ ^]^ By comparison, sharp H3K4me3 and H2A.Z are acquired at active and poised promoters during mouse ZGA, whereas bivalency is not established at these poised promoters until the morula stage. These observations indicate that ZGA occurs coordinately with lineage commitment in lower animal embryos, whereas these two biological processes are relatively separate in mammals. Considering that H2A.Z accumulates before ZGA in *Drosophila* and zebrafish embryos,^[^ ^42^, ^43^ ^]^ but accumulates at the ZGA stage in mammalian embryos, we speculate that H2A.Z plays a distinct role in future gene expression between mammals and lower animals. It will be interesting to characterize the TFs and chromatin remodelers orchestrating ZGA and lineage commitment in different species.

## 4 Conclusion

In sum, our findings regarding the hierarchical accumulation of H2A.Z explain its dual roles to regulate gene expression and histone modifications in early embryos. These data may provide the cornerstones for future investigations of mammalian ZGA initiation and lineage commitment during preimplantation development.

## 5 Experimental Section

### Mouse Oocyte and Embryo Collection

Specific‐pathogen‐free mice were housed in the animal facility of Huazhong Agricultural University, Wuhan, China. All animal procedures complied with the Animal Care and Use Committee of Huazhong Agriculture University (Approval number: HZAUMO‐2019‐078). To collect early embryos, B6D2F1 (C57BL/6 × DBA/2) female mice (8 weeks old) were superovulated by injection with pregnant mare serum gonadotropin (PMSG, 10 IU) (Ningbo Second Hormone Factory, China), followed by injection of human chorionic gonadotropin (hCG, 5 IU) (Ningbo Second Hormone Factory, China) 48 h later. The superovulated female mice were mated with B6D2F1 male mice, and were sacrificed to collect zygotes. These IVO embryos were then cultured in G1‐plus medium (10132, Vitrolife) at 37°C under 5% CO_2_ in air, and harvested as zygotes (pronuclear stage 5), early 2‐cell, 2‐cell, 4‐cell, 8‐cell embryos, morulae and blastocysts at 26 h, 30 h, 43–44 h, 55 h, 68 h, 78 h, and 94 h post hCG injection, respectively. ICM and TE isolation from blastocysts was performed as previously described.^[^ ^14a^ ^]^ MII oocytes were collected from superovulated C57BL/6 female mice (8 weeks old) at 12–13 h post hCG injection. For IVF, cauda epididymis was collected from DBA/2 male mice (10 weeks old) to squeeze out and incubate the semen in HTF medium (MR‐070, Sigma) for capacitation at 37°C for 1 h. MII oocytes collected from C57BL/6 female mice were incubated with activated sperm in HTF medium supplemented with 10 mg mL^−1^ bovine serum albumin (BSA, Sigma) at 37°C for 4 h, and cultured in G1‐plus medium. For parthenogenetic activation (PA), MII oocytes collected from C57BL/6 female mice were incubated in Ca^2+^‐free CZB medium^[^ ^44^ ^]^ supplemented with 1 × 10^−2^ M SrCl_2_ (Sigma) and 5 mg mL^−1^ of cytochalasin B (Sigma) for 4 h, and cultured in G1‐plus medium. IVF and PA embryos at the 2‐cell stage were harvested at 32 h post insemination and chemical activation, respectively.

### Pig Embryo Collection

Pig oocyte in vitro maturation and IVF were performed as previously described.^[^ ^45^ ^]^ IVF embryos at the morula stage were harvested at day 5 (120 h) post insemination.

### Microinjection

Small interference RNAs (siRNAs) against mouse *H2A.Z*, *Anp32e*, *Srcap* and porcine *H2A.Z* were designed and synthesized for three pairs by GenePharma, Shanghai, China. All three pairs of siRNAs for each gene were diluted and mixed in nuclease‐free water with a working concentration of 2 × 10^−5^ M. Mouse zygotes at the pronuclear stage 3 (20–22 h post hCG injection) were injected with ≈10 pL of siRNAs using a PiezoXpert micromanipulator (Eppendorf), and cultured in G1‐plus medium at 37°C under 5% CO_2_ in air. The injected embryos were observed at 44 h, 55 h, 68 h, 78 h, and 94 h post hCG injection to summarize the developmental phenotypes, or immediately transferred into the fallopian tubes of pseudopregnant Kunming mice with a mouth pipette. The recipients were sacrificed to collect the 5.5 day embryos. Porcine putative zygotes at 6 h post insemination were injected with ≈10 pL of siRNAs using FemtoJet 4i microinjector (Eppendorf), and cultured in PZM‐3 medium^[^ ^45^ ^]^ at 38.5°C under 5% CO_2_ in the air. The injected embryos were observed on day 2 (48 h), day 3 (72 h), day 5 (120 h), day 7 (156 h) post insemination to summarize the developmental phenotypes. All siRNA sequences are listed in Table S5, Supporting Information.

### Quantitative PCR

Total RNA isolation from 30 embryos or three 5.5 day embryos was performed by using RNAprep Pure Micro Kit (DP420, TIANGEN). cDNAs were synthesized by HiScript II Q RT SuperMix Kit plus gDNA wiper (R223‐01, Vazyme), and quantified by ChamQ Universal SYBR qPCR Master Mix (Q321‐02, Vazyme) on CFX96 Real‐Time PCR Detection System (Bio‐Rad). The results from noninjected control embryos or MII oocytes were set as 100% or 1, and were normalized to the internal control mouse gene *Gapdh* ^[^ ^44^ ^]^ or porcine gene *GADPH* ^[^ ^45^ ^]^ as previously described. Data were shown as the fold change (FC) = 2^−ΔΔ^ *^Ct^* mean ± standard deviation (SD). All primer sequences are listed in Table S5, Supporting Information.

### Immunofluorescence

Oocytes and embryos were fixed in 4% (w/v in PBS) paraformaldehyde (PFA, Sigma) for 1 h. After three washes in 0.05% (w/v in PBS) polyvinyl alcohol (PVA, Sigma), samples were permeabilized in 0.5% (v/v in PBS) Triton X‐100 (Sigma) for 30 min, blocked in 5% (w/v in PBS) BSA for 2 h, and incubated with primary antibodies diluted in 5% BSA at 4°C overnight. Primary antibodies used for immunostaining were rabbit anti‐H2A.Z (ab188314, Abcam; 1:200 dilution) and rabbit anti‐H3K4me3 (ab8580, Abcam; 1:500 dilution). After three washes in 0.05% PVA, samples were incubated with secondary antibodies diluted in 5% BSA for 1 h. Secondary antibodies used for immunostaining were Dylight 488/549 goat antirabbit IgG (A23220/A23320, Abbkine; 1:500 dilution). After another three washes, samples were mounted on glass slides with a drop of anti‐fade mounting medium containing 4,6‐diamidino‐2‐phenylindole (DAPI) (P0131, Beyotime). Fluorescence was detected and captured under a confocal microscope (LSM 800, Zeiss). All steps were performed at room temperature unless stated otherwise. Fluorescence intensity was determined by using ImageJ software (version1.48) as previously described.^[^ ^45^ ^]^ Cell number in embryos was counted as the number of nuclei stained blue by DAPI.

### Cell Colony Formation

Mouse embryonic stem cell (ESC) colony was derived as previously described with some modifications.^[^ ^46^ ^]^ Briefly, early blastocysts (88–90 h post hCG injection) were incubated in 0.5% (w/v in PBS) pronase E (Sigma) to remove zona pellucidae. One blastocyst was seeded on mitomycin‐C‐treated MEFs per 96‐well and cultured in ESC derivation medium at 37°C under 5% CO_2_ in the air. ESC derivation medium contains KnockOut DMEM (10829018, Gibco) supplemented with 15% (v/v) KnockOut serum replacement (10828028, Gibco), MEM nonessential amino acids solution (11140050, Gibco), GlutaMAX supplement (35050061, Gibco), 2‐mercaptoethanol (ES‐007‐E, Millipore), nucleosides (ES‐008‐D, Millipore), leukemia inhibitory factor (LIF, ESG1107, Millipore), 1 × 10^−6^ M PD0325901 (PZ0162, Sigma) and 3 × 10^−6^ M CHIR99021 (SML1046, Sigma). ESC colony was observed after 7 day culture, and could be digested and passaged using 0.05% (w/v) trypsin‐EDTA (25300054, Gibco). Mouse trophoblast stem cell (TSC) colony was derived according to another protocol.^[^ ^47^ ^]^ Briefly, one zona‐free early blastocyst was also seeded on MEF feeder per 96‐well and cultured in TSC derivation medium at 37°C under 5% CO_2_ in the air. TSC derivation medium contains RPMI 1640 (11875101, Gibco) supplemented with 20% (v/v) fetal bovine serum (FBS, Hyclone), sodium pyruvate (11360070, Gibco), 2‐mercaptoethanol, L‐Glutamine (25030081, Gibco), 1/1000 FGF4 stock (F2278, Sigma), 1/1000 heparin stock (H3149, Sigma). TSC colony was observed after 5 day culture. Colony forming rates of ESCs and TSCs were calculated as the ratio of the cell colony number at indicated timing to the total number of blastocysts used for this assay.

### RNA‐Seq Library Generation and Sequencing

The RNA‐seq libraries were generated using the Smart‐seq2 protocol as previously described with some modifications.^[^ ^48^ ^]^ Briefly, 15 embryos from *H2A.Z* KD and control groups were harvested at the 2‐cell, morula, and blastocyst stages. After zona pellucidae removal in 0.5% pronase E and three washes in 0.05% PVA, embryos were transferred into 4 µL lysis buffer containing Triton X‐100, oligo‐dT primer, dNTP (Thermo Fisher Scientific) and RNase inhibitor (Takara). After 72°C incubation for 3 min, 5.7 µL reverse transcription mix (100 U SuperScript II reverse transcriptase (18064014, Thermo Fisher Scientific), 1 × Superscript II first‐strand buffer, 5 × 10^−3^ m DTT, 1 M betaine, 6 × 10^−3^ m MgCl_2_, 1 × 10^−6^ m TSO, 10 U RNase inhibitor) was added into sample lysis buffer, followed by 42°C incubation for 90 min to obtain cDNA. Next, cDNA was preamplification for 16—18 cycles by using KAPA HiFi HotStart ReadyMix (KK2601, Roche) and IS PCR primers, followed by PCR purification by AMPure XP beads (A63881, Beckman). Finally, 1 ng of amplified cDNA was fragmented and the RNA‐seq library was constructed by using TruePrep DNA Library Prep Kit (TD502, Vazyme) according to the manufacturer's instructions. Paired‐end 150‐bp sequencing was performed on the Illumina HiSeq X‐Ten or NovaSeq 6000 system.

### ULI‐NChIP‐Seq Library Generation and Sequencing

ULI‐NChIP‐seq was performed as previously described with some modifications.^[^ ^13^ ^]^ For each immunoprecipitation reaction, ≈500 cells of MII oocytes, embryos, ICM, TE, and ESCs were harvested. After zona pellucidae removal in 0.5% pronase E and three washes in 0.05% PVA, mouse oocytes and embryos were incubated in Ca^2+^‐free CZB medium at 37°C for 5 min, followed by gentle pipetting to remove polar bodies. The zona pellucidae of porcine morula was removed by 0.25% pronase E. Embryos and cells were then added into 20 µL Nuclear Isolation buffer and 30 µL 10 U mL^−1^ MNase Master mix to digest chromatin for 7 min at 25°C, and added 5.5 µL 1 × 10^−1^ M EDTA and 5.5 µL 1% (v/v) Triton X‐100 plus 1% (w/v) deoxycholate solution to finish digestion. Next, samples were incubated with antibody‐bead complexes in 130 µL Complete Immunoprecipitation buffer overnight at 4 °C. These complexes were formed by preincubating 11 µL Dynabeads Protein G (10003D, Invitrogen) with 2 µg antibody of H2A.Z (ab188314, Abcam; ab4174, Abcam; ab150402, Abcam; 07–594, Millipore), H3K4me3 (ab8580, Abcam), H3K27me3 (07–449, Millipore) and Pol II (61668, Active Motif) in Complete Immunoprecipitation buffer for 6 h at 4°C. After two washes with 200 µL Low Salt Wash buffer and 200 µL High Salt Wash buffer, chromatin was dissolved in 100 µL ChIP Elution buffer for 2 h at 65°C. DNA was then extracted by 100 µL phenol‐chloroform‐isoamyl alcohol (25:24:1) (77 617, Sigma), and used for library generation by using KAPA HyperPrep Kit (KK8504, Roche) following the manufacturer's instructions. Paired‐end 150‐bp sequencing was performed on Illumina HiSeq X‐Ten or NovaSeq 6000 system.

### RNA‐Seq Data Processing

All RNA‐seq data were aligned to the mouse reference genome mm10 or pig reference genome susScr11 using STAR (version 2.7.3a). FPKM (fragments per kilobase of transcript per million mapped reads) values of genes were quantified using Stringtie (version 2.1.4). DEGs were identified using DESeq2 (version 1.30.1) based on the reads count file obtained by featureCounts (version 1.6.2). Genes with an absolute fold change (FC) >2 and *P* adjusted <0.05 were considered as significant DEGs. Genes with FPKM values >1 or <0.1 in each sample were termed active genes or inactive genes, respectively. Principal component analysis of RNA‐seq was performed using the plotPCA function of DESeq2 package in *R* (version 4.0.2).

### ChIP‐Seq Data Processing

All reads were mapped to the mouse reference genome mm10 or pig reference genome susScr11 using Bowtie2 (version 2.4.1). The SAMtools (version 1.9) was used to remove low‐quality reads (MAPQ <30) and Picard (version 2.23.9) was used to remove PCR duplicates. MACS2 (version 2.2.7.1) software calls ChIP‐seq peaks with the parameters “–nolambda –nomodel –broad”. The quality of ChIP‐seq data produced by different antibodies was evaluated as RelCC, SSD, RiP% by using ChIPQC (version 1.26.0) Bioconductor package. Normalized RPKM (reads per kilobase of bin per million mapped reads) bigwig files were generated by bamCoverage subcommand in deepTools (version 3.5.0) and the tracks were visualized with Integrative Genomics Viewer (IGV, version 2.6.2). To minimize the batch and cell type variations, ChIP‐seq enrichment at promoters (TSS ± 1 kb) were further *Z*‐score normalized among all promoters as previously reported.^[^ ^15c^ ^]^ Heatmaps and average profiles were created using Ngsplot (version 2.63) and normalized to RPM (reads per million mapped reads). Enrichment of H2A.Z peaks at promoter, gene body, distal, and repeat regions was calculated using observed versus expected probability as previously reported.^[^ ^14b^ ^]^ Differentially H2A.Z‐enriched peaks in ChIP‐seq data between IVF and PA 2‐cell embryos were identified using DiffBind (version 3.0.14) software. Peaks with FC >2 and false discovery rate <0.05 were considered as significantly different H2A.Z peaks.

### Identification of Gained, Lost and Maintained Peaks

To investigate the H2A.Z dynamics in early embryos, H2A.Z peaks at one stage that were not overlapping with H2A.Z peaks at the previous stage were defined as “gain” H2A.Z peaks at this stage. H2A.Z peaks at one stage that were not overlapping with the H2A.Z peaks at the next stage were defined as “loss” H2A.Z peaks at this stage. H2A.Z peaks at one stage that were overlapping with the H2A.Z peaks at the previous stage were defined as “maintain” H2A.Z peaks at this stage. Overlapping H2A.Z peaks were determined by the intersect function in BEDTools (version 2.27) package with the default settings.

### Genomic Distribution of H2A.Z Peaks

To identify the genomic distribution of H2A.Z peaks in early embryos, these peaks were annotated with the priority order (promoter > exon > intron > intergenic) using ChIPseeker (version 1.26.2) when a single peak spanned more than two genomic features.

### Clustering Analysis

RPKM values were calculated using a 5‐kb sliding window to compare genome‐wide ChIP‐seq enrichment among samples, or using promoter regions (TSS ± 1 kb) to compare promoter ChIP‐seq enrichment among samples. Hierarchical clustering was performed in *R* by stats package and hclust function with RPKM values via Pearson's correlations. *k*‐means clustering was used to classify all gene promoters into four clusters according to the different H2A.Z dynamics in early embryos.

### Gene Ontology Analysis

GO analysis for four gene clusters and DEGs in H2A.Z‐depleted morula was performed in *R* by clusterProfiler (version 3.18.1) package and enrichGO function.

### Allele‐Specific ChIP‐Seq Analysis

All H2A.Z ChIP‐seq reads were aligned to the genomes of the C57BL/6 and DBA/2 strains separately using Bowtie2. Single nucleotide polymorphism (SNP) tables for C57BL/6 and DBA/2 were downloaded from the Sanger Institute (http://www.sanger.ac.uk/science/data/mouse‐genomes‐project). Uniquely mapped reads were input into SNPsplit (version 0.3.2) software to determine the allele‐specific origin for the ChIP‐seq data. H2A.Z ChIP‐seq peaks covered by at least 10 SNP‐trackable reads were considered as allele‐specific peaks. To identify allele‐biased H2A.Z peaks at maternal and paternal genomes, each H2A.Z peak was subjected to a binomial exact test with the null hypothesis that both alleles were equally enriched. The origin of allele‐specific peaks was measured as the total number of reads mapped on the paternal genome divided by the total number of paternal and maternal reads for each peak: allelic ratio = paternal reads/(paternal + maternal) reads. Peaks showing allelic ratio ≥0.85 and *P* adjusted <0.05 were considered paternal biased; Peaks showing allelic ratio ≤0.15 and *P* adjusted <0.05 were considered maternal biased.

### Identification of Promoter H2A.Z or H3K4me3 Peak Types

Genes with RPKM values of promoter (TSS ± 1 kb) H2A.Z or H3K4me3 enrichment ≤1 in each sample were termed “No” enrichment, and the left genes (RPKM >1) were divided into three tertiles as “Low”, “Medium”, “High” enrichment depending on their promoter H2A.Z or H3K4me3 enrichment. Within Ngsplot, normalized H2A.Z or H3K4me3 ChIP‐seq bam files were used to generate average scores from −500 to −100 bp (−1 Nuc), −20 to 20 bp (TSS), and 100 to 500 bp (+1 Nuc) windows around TSS and ±1 nucleosome regions. To identify the peak types of promoter H2A.Z or H3K4me3, the following formula was used:

(1) $$K=\log_{2}\left(\frac{-1\text{Nuc}}{\mathrm{TSS}}\right)\times \log_{2}\left(\frac{+1\text{Nuc}}{\mathrm{TSS}}\right)$$

Promoters with strong H2A.Z or H3K4me3 enrichment (RPKM >1) were quantified as *K* values to define their peak types: “Double” type, *K* >0; “Single” type, *K* ≤0. Promoters with weak H2A.Z or H3K4me3 enrichment (RPKM ≤1) were defined as “No” type. The number of CpG at promoters (TSS ± 1 kb) with these three H2A.Z peak types was calculated using BEDTools.

### Motif Enrichment Analysis

To find the enrichment of transcription factor binding sites in H2A.Z‐enriched regions, findMotifsGenome.pl from HOMER program (version 4.11) was used. Motifs with known matches in HOMER database were selected.

### Identification of H3K27me3 Established de novo in ICM or Inherited from Oocytes

Genes with RPKM values of promoter (TSS ± 1 kb) H3K27me3 enrichment >1 in ICM were extracted. The ratio of H3K27me3 enrichment at each gene promoter in MII oocytes to 2‐cell/8‐cell embryos was then calculated. Genes with ratios >1 were considered as de novo H3K27me3 establishment occurs in ICM. Genes with ratios <0.5 were considered as maternal H3K27me3 inherits into early embryos.

### Identification of PcG Target Genes

Genes marked by H3K27me3 at their promoters (TSS ± 5 kb) in mouse ESCs were termed as PcG target genes. Enrichment of H2A.Z‐marked genes or DEGs that were PcG target genes was calculated using observed versus expected probability as previously reported.^[^ ^15c^ ^]^

### Amino Acid Sequence Alignment

Amino acid sequences and their accession numbers of 14 mammals were collected from the National Center for Biotechnology Information database (https://www.ncbi.nlm.nih.gov/), and aligned by using DNAMAN software (version 6.0.3.99) with default settings.

### Statistical Analysis

Statistical data were presented as the mean ± SD in bar charts. For boxplots, middle lines indicated the median, the boxes indicated the 25^th^/75^th^ percentiles, and the whiskers indicated 1.5× interquartile range (IQR). ULI‐NChIP‐seq was repeated twice. Except for the ULI‐NChIP‐seq, all experiments were repeated at least three times. *P* values were determined by a two‐sided Wilcoxon‐Mann‐Whitney test in *R*, or a two‐sided Student's *t*‐test using GraphPad Prism (version 8.0.1). Significant differences were shown with *, **, *** for indicating *P* <0.05, 0.01, and 0.001, respectively. n.s. denotes no significance.

## Conflict of Interest

The authors declare no conflict of interest.

## Supporting information

Supporting Information

## Data Availability

The data that support the findings of this study are openly available in NCBI Gene Expression Omnibus and CNCB Genome Sequence Archive at https://www.ncbi.nlm.nih.gov/geo/query/acc.cgi?acc=GSE188590; https://ngdc.cncb.ac.cn/gsa/s/HE6w7s8c; https://ngdc.cncb.ac.cn/gsa/s/jnXowulD. These data were derived from the following resources available in the public domain: [GSE188590], https://www.ncbi.nlm.nih.gov/geo/query/acc.cgi?acc=GSE188590; [CRA004237], https://ngdc.cncb.ac.cn/gsa/s/HE6w7s8c; [CRA003606], https://ngdc.cncb.ac.cn/gsa/s/jnXowulD.
