# Supplementary material for: Hierarchical Accumulation of Histone Variant H2A.Z Regulates Transcriptional States and Histone Modifications in Early Mammalian Embryos
Source: Adv Sci (Weinh). 2022 Jun 19;9(23):2200057. doi: 10.1002/advs.202200057 (PMC9376818; doi:10.1002/advs.202200057)
Supplement: Supplementary file 1 — Supporting Information [file ADVS-9-2200057-s001.pdf]

ADVANCED SCIENCE
Open Access

Supporting Information

for *Adv. Sci.*, DOI 10.1002/advs.202200057

Hierarchical Accumulation of Histone Variant H2A.Z Regulates Transcriptional States and Histone Modifications in Early Mammalian Embryos

*Xin Liu, Jingjing Zhang, Jilong Zhou, Guowei Bu, Wei Zhu, Hainan He, Qiaoran Sun, Zhisheng Yu, Wenjing Xiong, Liyan Wang, Danya Wu, Chengli Dou, Longtao Yu, Kai Zhou, Shangke Wang, Zhengang Fan, Tingting Wang, Ruifeng Hu, Taotao Hu, Xia Zhang and Yi-Liang Miao**

Supporting Information

Title

Hierarchical Accumulation of Histone Variant H2A.Z Regulates Transcriptional States and Histone Modifications in Early Mammalian Embryos

*Xin Liu, Jingjing Zhang, Jilong Zhou, Guowei Bu, Wei Zhu, Hainan He, Qiaoran Sun, Zhisheng Yu, Wenjing Xiong, Liyan Wang, Danya Wu, Chengli Dou, Longtao Yu, Kai Zhou, Shangke Wang, Zhengang Fan, Tingting Wang, Ruifeng Hu, Taotao Hu, Xia Zhang, and Yi-Liang Miao**

Supporting Tables

Table S1. RNA-seq and ChIP-seq experiment summary.

Table S2. H2A.Z peak types in 2-cell embryos, 8-cell embryos, ICM and ESC.

Table S3. Differentially expressed genes between control and *H2A.Z* KD embryos at the 2-cell, morula and blastocyst stages.

Table S4. Hierarchical H2A.Z transition in mouse control, *H2A.Z* KD, *Srcap* KD and *Anp32e* KD embryos at the morula stage.

Table S5. List of quantitative PCR primers and small interference RNA.

Supporting Figures

Figure S1-S8

Supporting References

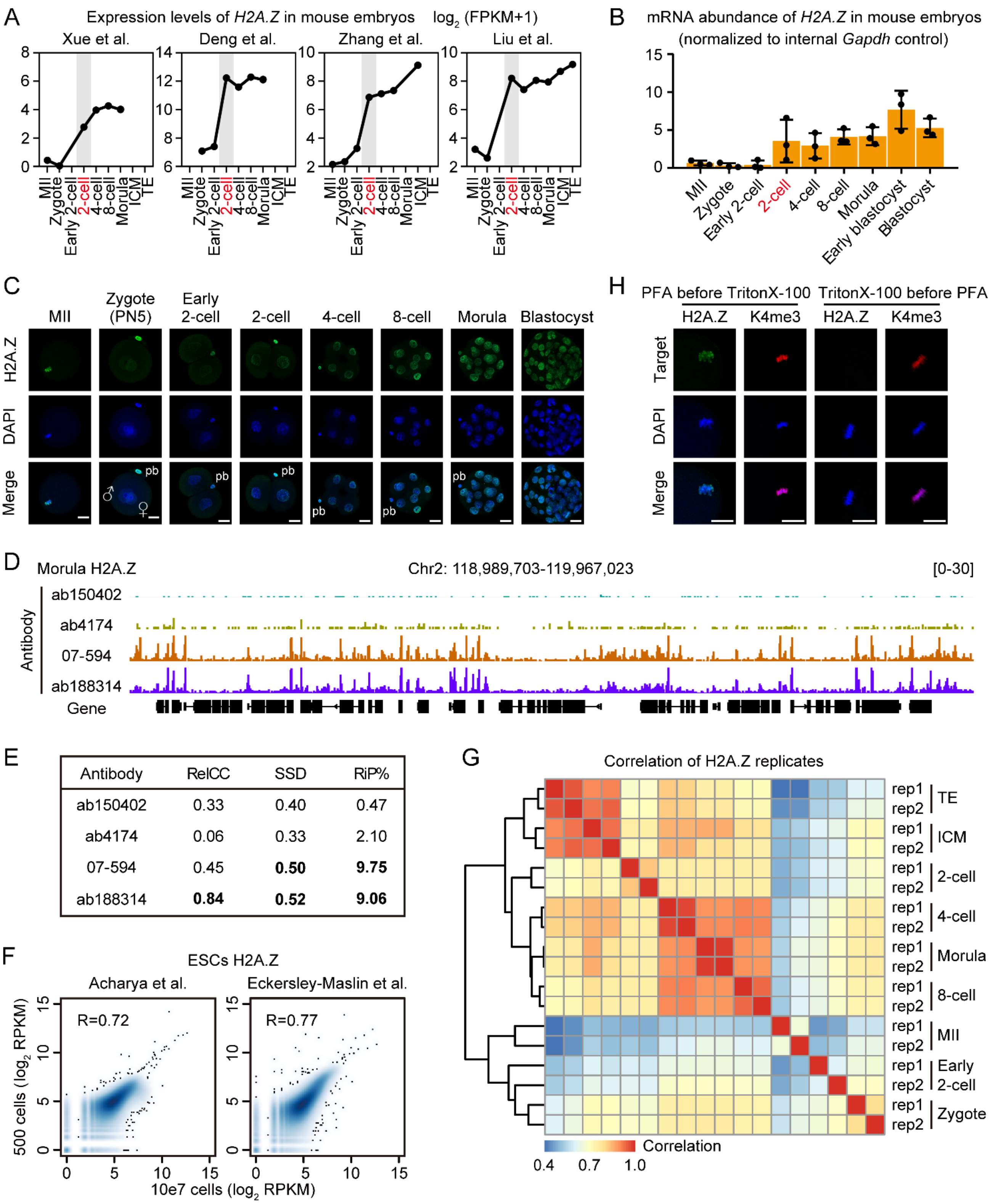

Antibody	RelCC	SSD	RiP%
ab150402	0.33	0.40	0.47
ab4174	0.06	0.33	2.10
07-594	0.45	**0.50**	**9.75**
ab188314	**0.84**	**0.52**	**9.06**

Figure S1. Validation of H2A.Z ChIP-seq data in mouse MII oocytes and early embryos. A) Line graphs showing the expression levels of *H2A.Z* in mouse metaphase II (MII) oocytes and early embryos by RNA sequencing (RNA-seq). RNA-seq data are from previous publications.[1]

Developmental stage marked in red and gray shades indicates the corresponding zygotic genome activation (ZGA) stage. FPKM, fragments per kilobase of transcript per million mapped reads. B) Bar charts showing the mRNA abundance of *H2A.Z* in MII oocytes and early embryos by quantitative PCR (qPCR). Error bars represent the standard deviation (SD) in three biological replicates. C) Immunostaining of H2A.Z (green) and DNA (blue) in MII oocytes and early embryos. One representative image from three independent experiments is shown. PN5, pronuclear stage 5; pb, polar body. Scale bar, 20 μm. D) Genome browser snapshot of H2A.Z enrichment in morulae by using different H2A.Z antibodies for ultra-low-input native chromatin immunoprecipitation and sequencing (ULI-NChIP-seq). Catalog numbers of each antibody are shown. Chr, chromosome. E) Quality assessment of ULI-NChIP-seq data by using different H2A.Z antibodies. RelCC, relative strand cross-correlation coefficient SSD; SSD, sum of standard deviations; RiP%, percentage of reads in peaks. Numbers in bold type indicate relatively good outputs. F) Scatter plots comparing the genome-wide H2A.Z enrichment (5-kb bins, n = 546,207) between ULI-NChIP-seq using 500 embryonic stem cells (ESCs) and conventional ChIP-seq using bulk ESCs. Pearson's correlation coefficients are shown on the top-left panel. H2A.Z ChIP-seq data using bulk ESCs are from previous publications.[2] RPKM, reads per kilobase of bin per million mapped reads. G) Heatmaps and hierarchical clustering showing the Pearson's correlations of genome-wide H2A.Z enrichment (5-kb bins) between replicates among stages (two biological replicates in each stage). H) Immunostaining of H2A.Z (green), H3K4me3 (red, K4me3) and DNA (blue) in MII oocytes with paraformaldehyde (PFA) fixation before permeabilization by Triton X-100 or in reverse. One representative image from three independent experiments is shown. Scale bar, 20 μm.

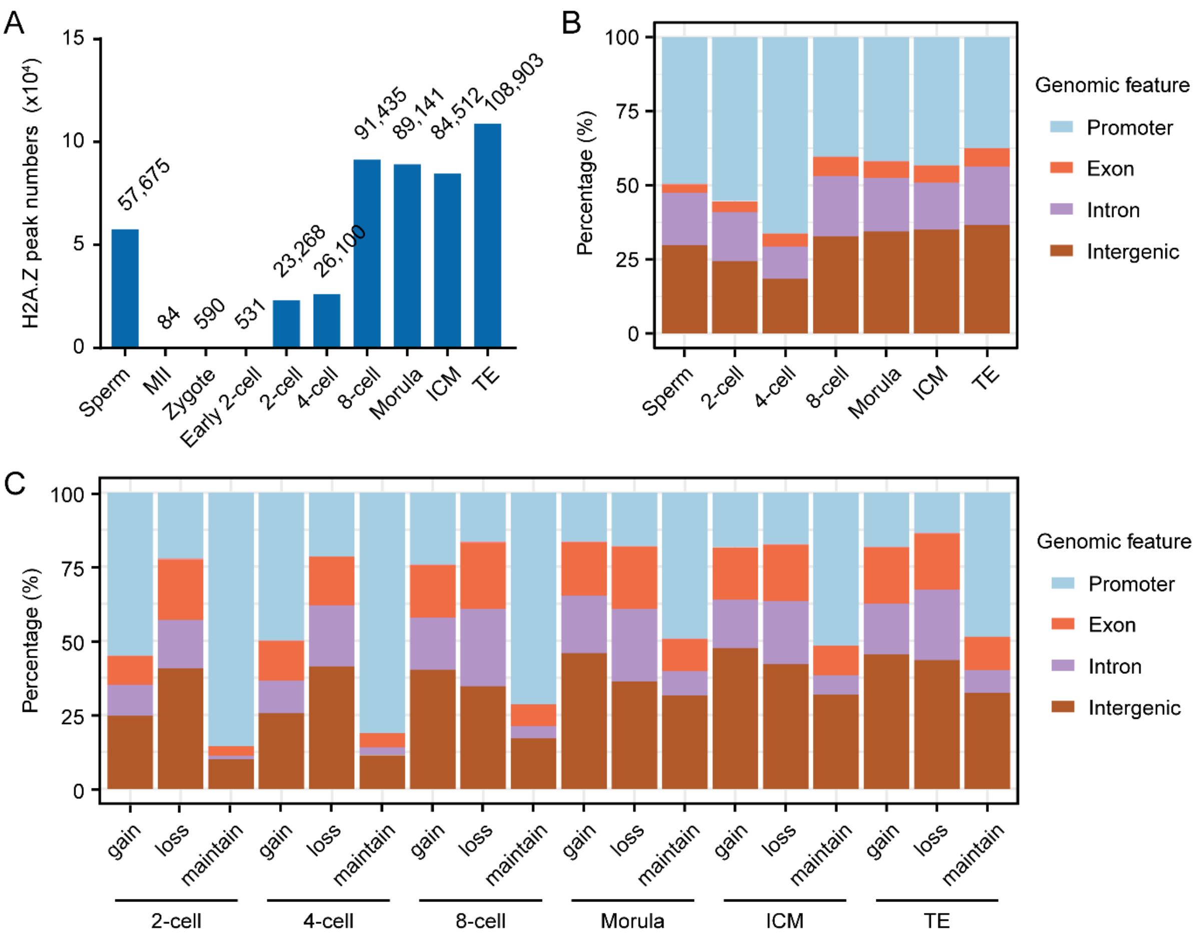

Figure S2. Genomic features of H2A.Z peaks in mouse sperm, MII oocytes and early embryos. A) Bar charts showing the total number of H2A.Z peaks detected in mouse sperm, MII oocytes and early embryos. H2A.Z ChIP-seq data in sperm are from a previous publication.[3] B) Percentage of total H2A.Z peaks that are located at promoter, exon, intron and intergenic regions in sperm and early embryos. C) Percentage of specific H2A.Z peaks that are located at promoter, exon, intron and intergenic regions in early embryos. These specific H2A.Z peaks are gained, lost and maintained at each stage by comparing to the previous stage.

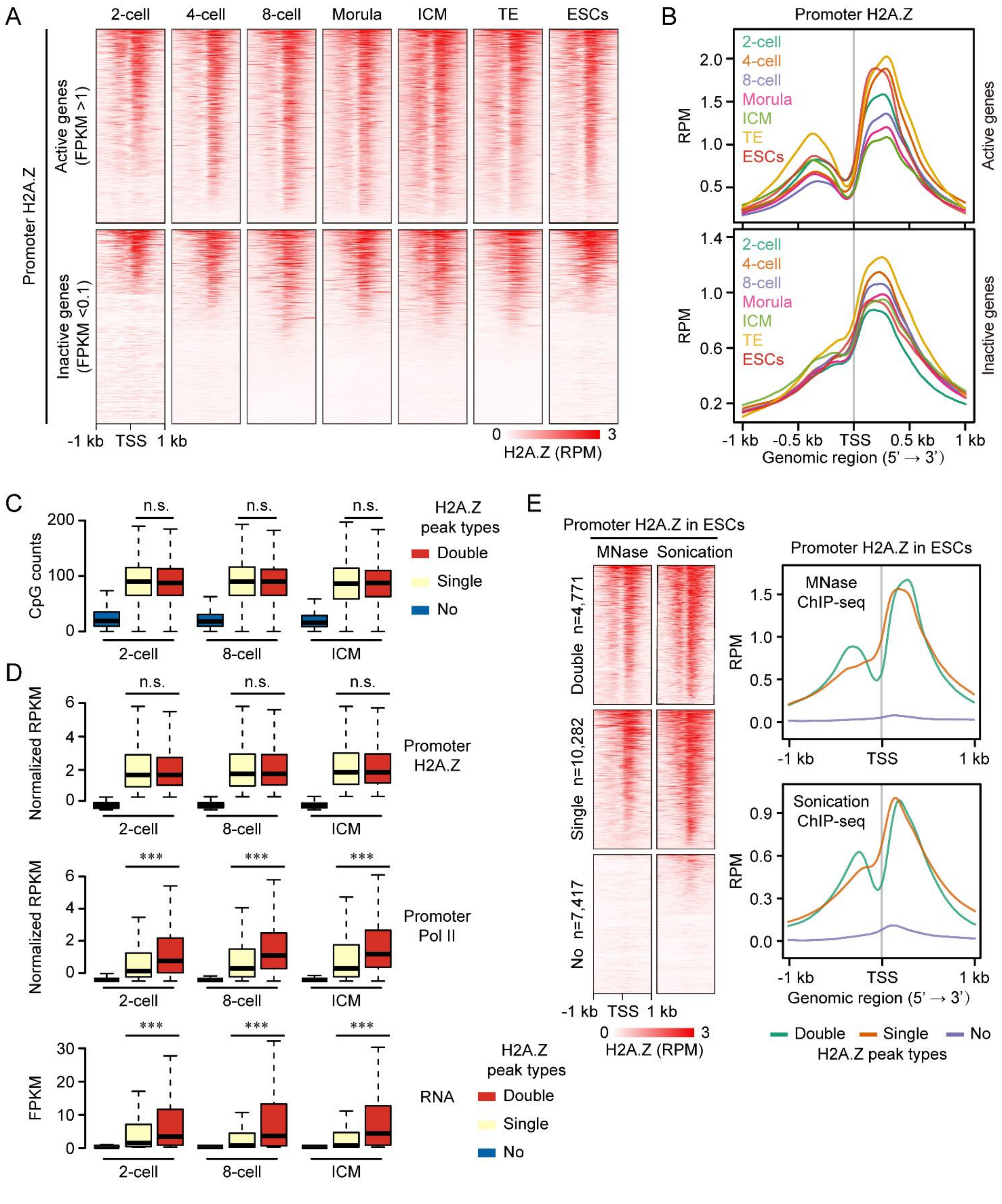

Figure S3. Relationship among H2A.Z, Pol II binding and gene expression. A) Heatmaps showing the H2A.Z enrichment at active (top) and inactive (bottom) gene promoters in mouse early embryos and ESCs. Each row represents a promoter region (TSS ± 1 kb) and is ordered descending by H2A.Z enrichment. RPM, read counts per million mapped reads. B) Profiles showing the average H2A.Z

enrichment at active (top) and inactive (bottom) gene promoters in mouse early embryos and ESCs. C) Boxplot comparing the CpG counts of promoters with three H2A.Z peak types in 2-cell embryos, 8-cell embryos and ICM. n.s., no significance; two-sided Wilcoxon-Mann-Whitney test. For boxplots, middle lines indicate the median, the boxes indicate the 25th/75th percentiles, and the whiskers indicate 1.5× interquartile range (IQR). D) Boxplots showing the promoter H2A.Z enrichment (top), RNA polymerase II (Pol II) enrichment (middle) and RNA levels (bottom) for genes with three H2A.Z peak types in 2-cell embryos, 8-cell embryos and ICM. Pol II ChIP-seq and RNA-seq data for early embryos are from previous publications.[1d, 4] ***P <0.001; n.s., no significance; two-sided Wilcoxon-Mann-Whitney test. For boxplots, middle lines indicate the median, the boxes indicate the 25th/75th percentiles, and the whiskers indicate 1.5× IQR. E) Heatmaps (left) and profiles (right) showing the promoter H2A.Z enrichment with three peak types generated by ULI-NChIP-seq and conventional ChIP-seq in ESCs. Chromatin are fragmented by MNase digestion in ULI-NChIP-seq and by sonication in conventional ChIP-seq. The numbers of genes with “Double”, “Single” and “No” H2A.Z peaks by ULI-NChIP-seq are shown. Each row in heatmaps represents a promoter region and is ordered descending by H2A.Z enrichment. H2A.Z ChIP-seq data by sonication are from a previous publication.[5]

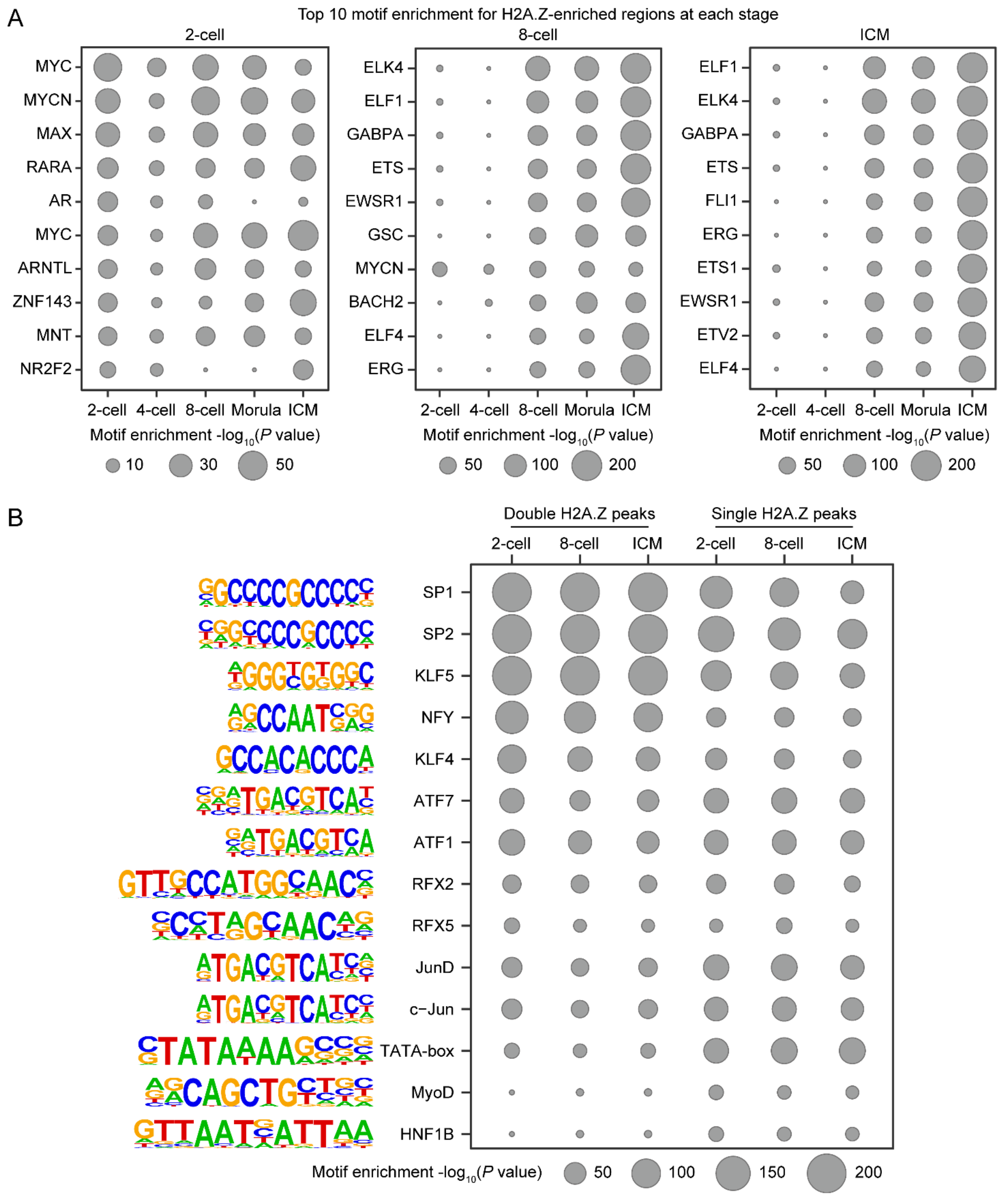

Figure S4. Motif analysis of H2A.Z-enriched promoters in mouse early embryos. A) Enrichment scores of top 10 transcription factor binding motifs for the H2A.Z peaks in mouse 2-cell embryos, 8-cell embryos and ICM. The point size represents the motif enrichment P value ($-\log_{10} P$ value). B) Enrichment scores of candidate transcription factor binding motifs for the “Double” and “Single”

types of H2A.Z peaks in mouse 2-cell embryos, 8-cell embryos and ICM. The point size represents the motif enrichment *P* value ($-\log_{10}$ *P* value).

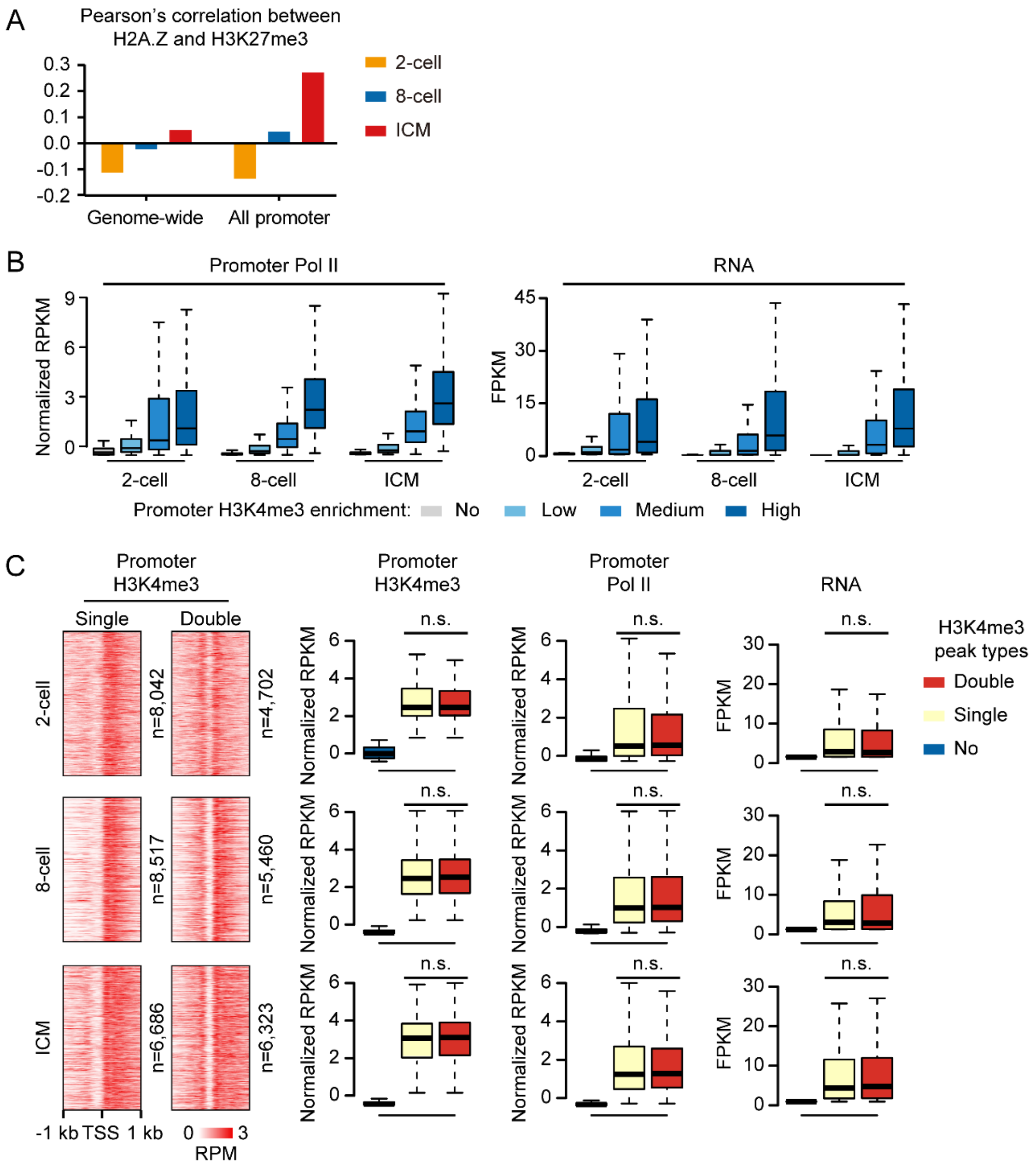

Figure S5. Different features between the peak types of H2A.Z and H3K4me3 in mouse early embryos. A) Bar charts showing the Pearson's correlations between H2A.Z and H3K27me3 enrichment in 2-cell embryos, 8-cell embryos and ICM on genome-wide (5-kb bins, n = 546,207) and at promoters (n = 22,470). B) Boxplots showing Pol II enrichment (left) and RNA levels (right) for four gene clusters in early embryos. Genes with weak promoter H3K4me3 enrichment (RPKM≤1) are extracted first, and the remaining genes are divided into three tertiles depending on promoter H3K4me3 enrichment. For boxplots, middle lines indicate the median, the boxes indicate the 25th/75th percentiles, and the whiskers indicate 1.5× IQR. C) Heatmaps showing the H3K4me3

enrichment in 2-cell embryos, 8-cell embryos and ICM at promoters with “Double” and “Single” H3K4me3 peaks. The numbers of genes with “Double” and “Single” H3K4me3 peaks in each sample are shown. Each row represents a promoter region and is ordered randomly. Boxplots showing the promoter H3K4me3 enrichment (left), Pol II enrichment (middle) and RNA levels (right) for genes with three H3K4me3 peak types in 2-cell embryos, 8-cell embryos and ICM. H3K4me3 ChIP-seq, Pol II ChIP-seq, and RNA-seq data for early embryos are from previous publications.[1d, 4, 6] n.s., no significance; two-sided Wilcoxon-Mann-Whitney test. For boxplots, middle lines indicate the median, the boxes indicate the $25^{th}/75^{th}$ percentiles, and the whiskers indicate 1.5× IQR.

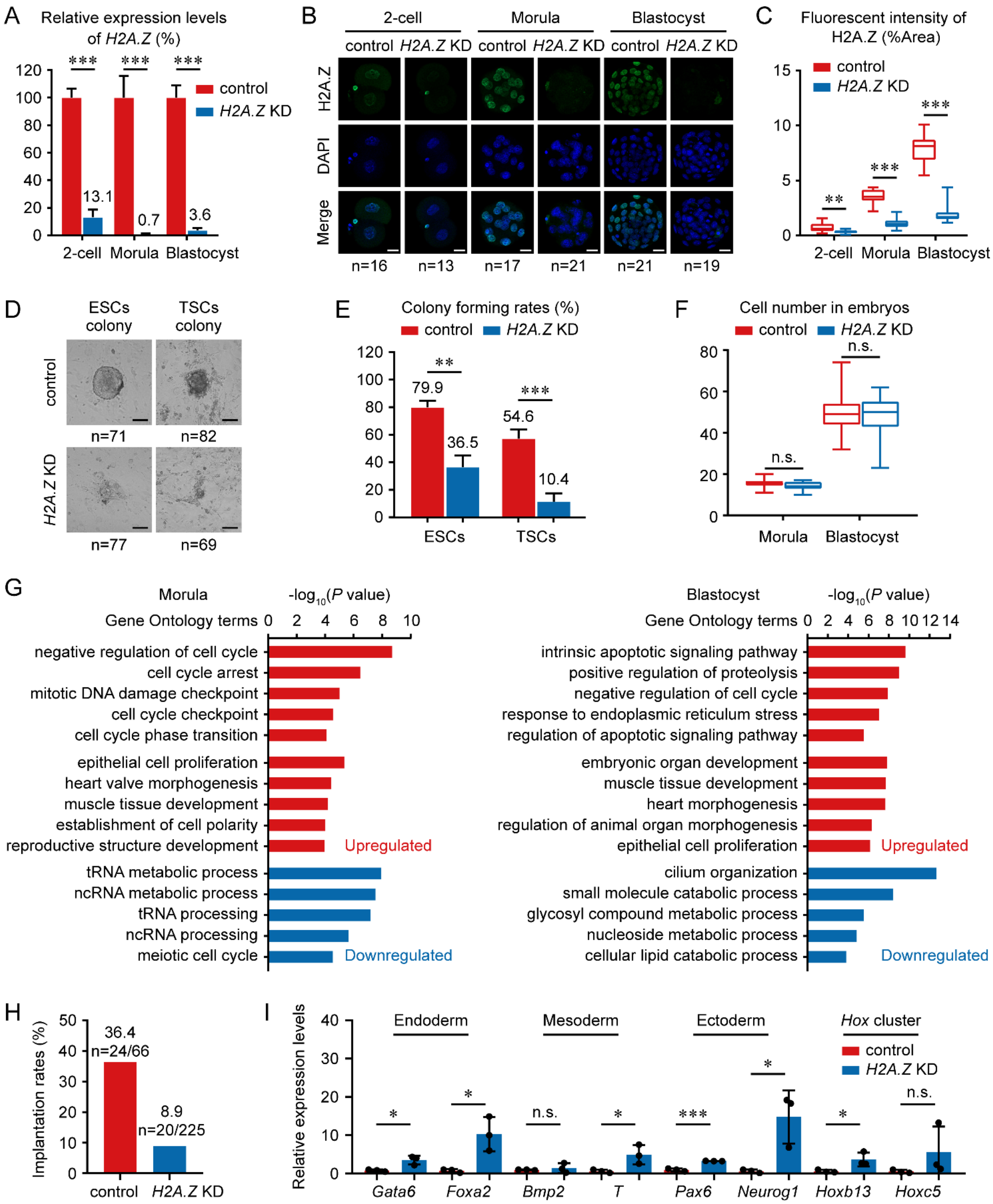

Figure S6. H2A.Z depletion in mouse early embryos. A) Bar charts showing the relative expression levels of *H2A.Z* in control and *H2A.Z* knockdown (KD) embryos at the 2-cell, morula and blastocyst stages. *H2A.Z* expression levels in control embryos are set as 100%, and the relative *H2A.Z*

expression levels in *H2A.Z* KD embryos are shown as percentages. Error bars represent the SD in three replicates. ***P <0.001; two-sided Student's t test. B) Immunostaining of H2A.Z (green) and DNA (blue) in control and *H2A.Z* KD embryos at the 2-cell, morula and blastocyst stages. One representative image and the numbers of embryos analyzed from three independent experiments are shown. Scale bar, 20 μm. C) Boxplots showing the fluorescent intensity of H2A.Z in control and *H2A.Z* KD embryos at the 2-cell, morula and blastocyst stages. ***P <0.001; **P <0.01; two-sided Student's t test. For boxplots, middle lines indicate the median, the boxes indicate the $25^{th}/75^{th}$ percentiles, and the whiskers indicate 1.5× IQR. D) Representative images of ESCs and trophoblast stem cells (TSCs) clones derived from control and *H2A.Z* KD blastocysts after 7-day and 5-day culturing, respectively. One representative image and the numbers of blastocysts used from three independent experiments are shown. Scale bar, 100 μm. E) Bar charts showing the colony forming rates for ESCs and TSCs by using control and *H2A.Z* KD blastocysts. Error bars represent the SD in three replicates. ***P <0.001; **P <0.01; two-sided Student's t test. F) Boxplots showing the cell number in control and *H2A.Z* KD embryos at the morula and blastocyst stages. n.s., no significance; two-sided Student's t test. For boxplots, middle lines indicate the median, the boxes indicate the $25^{th}/75^{th}$ percentiles, and the whiskers indicate 1.5× IQR. G) Bar charts showing the enriched Gene Ontology terms for upregulated and downregulated genes in *H2A.Z* KD embryos at the morula and blastocyst stages. H) Bar charts showing the implantation rates (the number of implantation site is divided by the total number of transferred zygotes) at day 5.5 when control (n = 66) and *H2A.Z* KD (n = 225) zygotes were transferred into the fallopian tubes of pseudopregnant Kunming mice. I) Bar charts showing the relative expression levels of representative lineage marker genes in control and *H2A.Z* KD embryos at day 5.5. Error bars represent the SD in three biological replicates. ***P <0.001; *P <0.05; n.s., no significance; two-sided Student's t test.

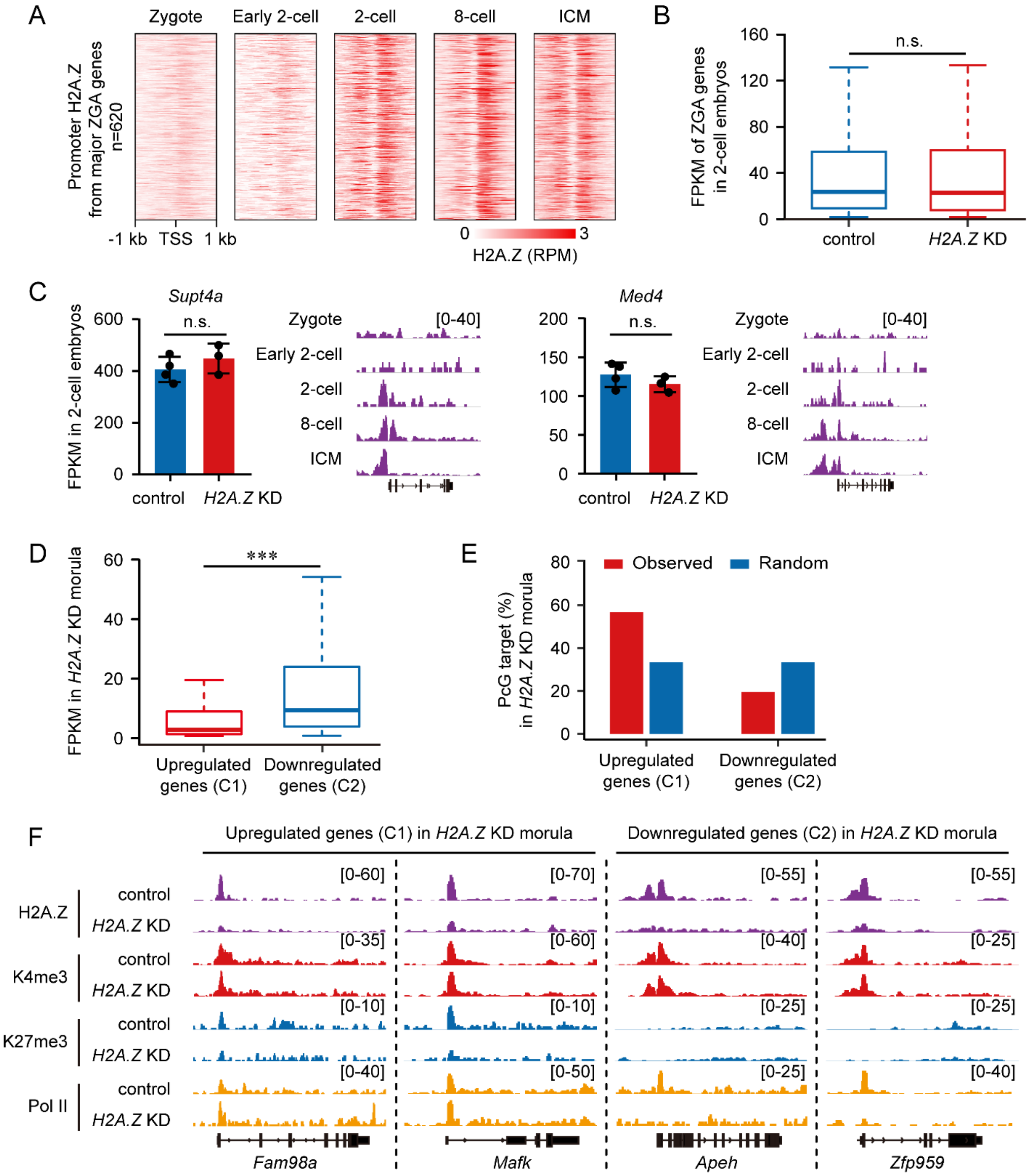

Figure S7. Aberrant transcriptome and epigenome in *H2A.Z* KD embryos. A) Heatmaps showing the H2A.Z enrichment at promoters of ZGA genes (n = 620)[7] in mouse early embryos. Each row represents a promoter region and is ordered randomly. B) Boxplots showing the expression levels of ZGA genes in control and *H2A.Z* KD embryos at the 2-cell stage. n.s., no significance; two-sided Wilcoxon-Mann-Whitney test. For boxplots, middle lines indicate the median, the boxes indicate the

25th/75th percentiles, and the whiskers indicate 1.5× IQR. C) Bar chart showing the expression levels of candidate ZGA genes (*Supt4a* and *Med4*) in control and *H2A.Z* KD embryos at the 2-cell stage. Error bars represent the SD in 3-4 biological replicates. n.s., no significance; two-sided Student's *t* test. Genome browser snapshot of H2A.Z enrichment in mouse early embryos near these two candidate ZGA genes. D) Boxplots showing the expression levels of H2A.Z-marked upregulated genes (C1, n = 330) and H2A.Z-marked downregulated genes (C2, n = 844) in control embryos at the morula stage. ***P <0.001; two-sided Wilcoxon-Mann-Whitney test. For boxplots, middle lines indicate the median, the boxes indicate the 25th/75th percentiles, and the whiskers indicate 1.5× IQR. E) Percentages of upregulated (C1) and downregulated (C2) genes in *H2A.Z* KD embryos at the morula stage that are polycomb group (PcG) target genes. A similar analysis for a set of random genes with the same size to the corresponding genes is performed as control. F) Genome browser snapshot of the enrichment of H2A.Z, H3K4me3, H3K27me3 and Pol II in control and *H2A.Z* KD embryos at the morula stage near upregulated genes (C1, *Fam98a* and *Mafk*) and downregulated genes (C2, *Apeh* and *Zfp959*).

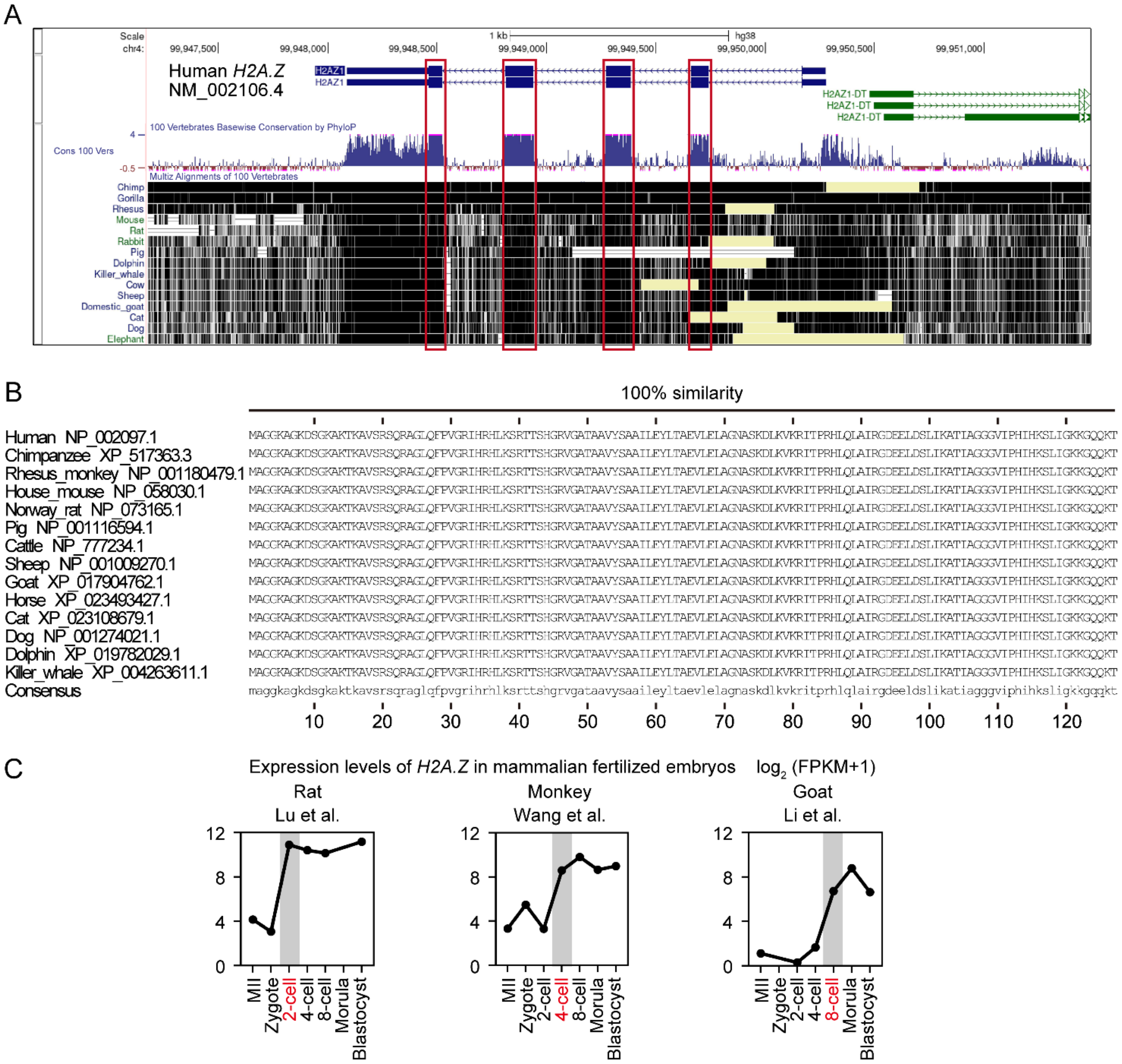

Figure S8. Conserved sequences of H2A.Z in different mammals. A) Genome snapshot on human GRCh38/hg38 assembly exhibits RefSeq genes, PhyloP basewise conservation score from 100 vertebrates, and multiz alignments of 15 mammals around human *H2A.Z* gene location. Red boxes highlight the highly conserved protein coding sequence of human *H2A.Z* in 15 mammals. B) Amino acid sequences of H2A.Z from 14 mammals. Sequences and their accession numbers are collected from National Center for Biotechnology Information (https://www.ncbi.nlm.nih.gov/), and aligned using DNAMAN software (version 6.0.3.99) with default settings. The sequence similarity is 100% for all H2A.Z proteins. C) Line graphs showing the expression levels of *H2A.Z* in rat, monkey, and caprine MII oocytes and early embryos by RNA-seq. RNA-seq data for rat,[8] monkey[9] and goat[10]

are from previous publications. Developmental stage marked in red and gray shades indicates the corresponding ZGA stage.

Supporting References

[1] a) Z. Xue, K. Huang, C. Cai, L. Cai, C. Y. Jiang, Y. Feng, Z. Liu, Q. Zeng, L. Cheng, Y. E. Sun, J. Y. Liu, S. Horvath, G. Fan, *Nature* **2013**, *500* (7464), 593; b) Q. Deng, D. Ramskold, B. Reinius, R. Sandberg, *Science* **2014**, *343* (6167), 193; c) B. Zhang, H. Zheng, B. Huang, W. Li, Y. Xiang, X. Peng, J. Ming, X. Wu, Y. Zhang, Q. Xu, W. Liu, X. Kou, Y. Zhao, W. He, C. Li, B. Chen, Y. Li, Q. Wang, J. Ma, Q. Yin, K. Kee, A. Meng, S. Gao, F. Xu, J. Na, W. Xie, *Nature* **2016**, *537* (7621), 553; d) W. Liu, X. Liu, C. Wang, Y. Gao, R. Gao, X. Kou, Y. Zhao, J. Li, Y. Wu, W. Xiu, S. Wang, J. Yin, W. Liu, T. Cai, H. Wang, Y. Zhang, S. Gao, *Cell Discov* **2016**, *2*, 16010.

[2] a) D. Acharya, S. J. Hainer, Y. Yoon, F. Wang, I. Bach, J. A. Rivera-Perez, T. G. Fazzio, *Cell Rep* **2017**, *19* (4), 671; b) M. A. Eckersley-Maslin, A. Parry, M. Blotenburg, C. Krueger, Y. Ito, V. N. R. Franklin, M. Narita, C. S. D'Santos, W. Reik, *Nat Struct Mol Biol* **2020**, *27* (8), 696.

[3] Y. H. Jung, I. Kremsky, H. B. Gold, M. J. Rowley, K. Punyawai, A. Buonanotte, X. Lyu, B. J. Bixler, A. W. S. Chan, V. G. Corces, *Mol Cell* **2019**, *75* (1), 154.

[4] B. Liu, Q. Xu, Q. Wang, S. Feng, F. Lai, P. Wang, F. Zheng, Y. Xiang, J. Wu, J. Nie, C. Qiu, W. Xia, L. Li, G. Yu, Z. Lin, K. Xu, Z. Xiong, F. Kong, L. Liu, C. Huang, Y. Yu, J. Na, W. Xie, *Nature* **2020**, *587* (7832), 139.

[5] C. C. Hsu, D. Zhao, J. Shi, D. Peng, H. Guan, Y. Li, Y. Huang, H. Wen, W. Li, H. Li, X. Shi, *Cell Discov* **2018**, *4*, 28.

[6] X. Liu, C. Wang, W. Liu, J. Li, C. Li, X. Kou, J. Chen, Y. Zhao, H. Gao, H. Wang, Y. Zhang, Y. Gao, S. Gao, *Nature* **2016**, *537* (7621), 558.

[7] S. J. Park, K. Shirahige, M. Ohsugi, K. Nakai, *Nucleic Acids Res* **2015**, *43* (Database issue), D771.

[8] X. Lu, Y. Zhang, L. Wang, L. Wang, H. Wang, Q. Xu, Y. Xiang, C. Chen, F. Kong, W. Xia, Z. Lin, S. Ma, L. Liu, X. Wang, H. Ni, W. Li, Y. Guo, W. Xie, *Sci Adv* **2021**, *7* (48), eabi6178.

[9] X. Wang, D. Liu, D. He, S. Suo, X. Xia, X. He, J. J. Han, P. Zheng, *Genome Res* **2017**, *27* (4), 567.

[10] Y. Li, J. Sun, Y. Ling, H. Ming, Z. Chen, F. Fang, Y. Liu, H. Cao, J. Ding, Z. Cao, X. Zhang, K. Bondioli, Z. Jiang, Y. Zhang, *Reprod Fertil Dev* **2020**, *32* (7), 714.
